# Lysine Ethylation by Histone Lysine Methyltransferases

**DOI:** 10.1002/cbic.201900359

**Published:** 2019-10-24

**Authors:** Abbas H. K. Al Temimi, Michael Martin, Qingxi Meng, Danny C. Lenstra, Ping Qian, Hong Guo, Elmar Weinhold, Jasmin Mecinović

**Affiliations:** ^1^ Institute for Molecules and Materials Radboud University Heyendaalseweg 135 6525 AJ Nijmegen The Netherlands; ^2^ Institute of Organic Chemistry RWTH Aachen University Landoltweg 1 52056 Aachen Germany; ^3^ Chemistry and Material Science Faculty Shandong Agricultural University Daizong Road No.61 Tai'an 271018 P.R. China; ^4^ Department of Biochemistry and Cellular and Molecular Biology University of Tennessee 1311 Cumberland Avenue Knoxville TN 37996 USA; ^5^ UT/ORNL Center for Molecular Biophysics Oak Ridge National Laboratory 1 Bethel Valley Road Oak Ridge TN 37830 USA; ^6^ Department of Physics, Chemistry and Pharmacy University of Southern Denmark Campusvej 55 5230 Odense Denmark

**Keywords:** epigenetics, enzymes, histones, molecular dynamics, transferases

## Abstract

Biomedicinally important histone lysine methyltransferases (KMTs) catalyze the transfer of a methyl group from *S*‐adenosylmethionine (AdoMet) cosubstrate to lysine residues in histones and other proteins. Herein, experimental and computational investigations on human KMT‐catalyzed ethylation of histone peptides by using *S*‐adenosylethionine (AdoEth) and *Se*‐adenosylselenoethionine (AdoSeEth) cosubstrates are reported. MALDI‐TOF MS experiments reveal that, unlike monomethyltransferases SETD7 and SETD8, methyltransferases G9a and G9a‐like protein (GLP) do have the capacity to ethylate lysine residues in histone peptides, and that cosubstrates follow the efficiency trend AdoMet>AdoSeEth>AdoEth. G9a and GLP can also catalyze AdoSeEth‐mediated ethylation of ornithine and produce histone peptides bearing lysine residues with different alkyl groups, such as H3K9meet and H3K9me2et. Molecular dynamics and free energy simulations based on quantum mechanics/molecular mechanics potential supported the experimental findings by providing an insight into the geometry and energetics of the enzymatic methyl/ethyl transfer process.

## Graphical abstract

**Transfer window**: Histone lysine methyltransferases, which catalyze the methylation of histones with *S*‐adenosylmethionine cosubstrate, have a limited capacity to catalyze ethylation of histones with *S*‐adenosylethionine (AdoEth) and *Se*‐adenosylselenoethionine (AdoSeEth) cosubstrates. Insights into the geometry and energetics of the enzymatic methyl/ethyl transfer process are reported.

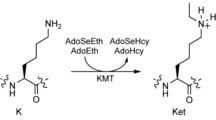

## Introduction

**Figure 1:**
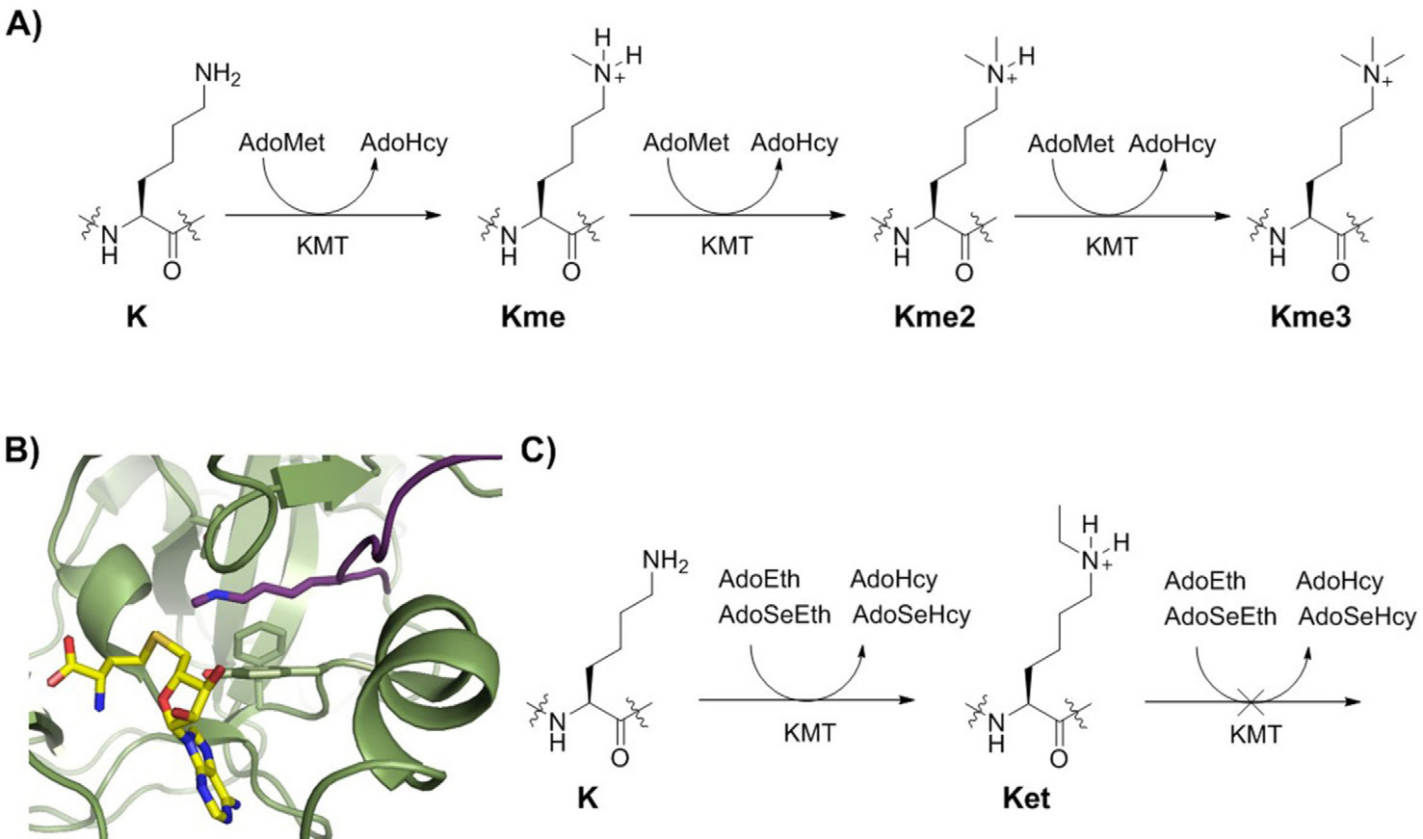
KMT‐catalyzed alkylation of histones. A) AdoMet‐mediated lysine methylation, leading to Kme, Kme2, and Kme3. B) View from a crystal structure of G9a‐like protein (GLP; green) in complex with H3K9me (violet) and *S*‐adenosylhomocysteine (AdoHcy; yellow; PDB ID: 3HNA). C) *S*‐Adenosylethionine (AdoEth)‐ or *Se*‐adenosylselenoethionine (AdoSeEth)‐mediated lysine ethylation, leading to Ket.

Histone proteins are subject to diverse post‐translational modifications (PTMs), including methylation, acetylation, crotonylation, phosphorylation, citrullination, and ubiquitination, which regulate the activity of human genes through epigenetic mechanisms.^1^ Methylation of lysine residues in unstructured histone tails is associated with both gene activation and repression, depending on the histone, methylation state, and methylation site.^2^ Histone lysine methylation is catalyzed by *S*‐adenosylmethionine (AdoMet)‐dependent histone lysine methyltransferases (KMTs) that install one (Kme), two (Kme2), or three (Kme3) methyl groups on the *N* ^*ϵ*^‐amino group of lysine (Figure 1 A).^3^ Histone lysine methylation is removed by flavin‐dependent lysine‐specific demethylases and Fe^II^/2‐oxoglutarate (2OG)‐dependent histone demethylases (KDMs),^4^ and recognized by a large number of *N* ^*ϵ*^‐methyllysine‐binding epigenetic reader proteins,^5^ which collectively spread the epigenetic landscape of post‐translational modifications.

Histone KMTs contain the conserved SET (Su(var)3–9, enhancer of zeste, trithorax) domain responsible for the enzymatic activity; DOT1L is the only member of the histone KMT family, known to date, that does not contain the SET domain.^6^ Structural analyses revealed that KMTs possess distinct binding pockets for AdoMet cosubstrate and histone substrate (Figure 1 B).6e In the ternary complex, the nucleophilic *N* ^*ϵ*^‐amino group of lysine is well aligned with the electrophilic methyl group of AdoMet for an efficient S_N_2 reaction that takes place in a narrow hydrophobic channel typically comprised of side chains of Tyr and Phe residues (Figure 1 B). The presence of Tyr and Phe in the active sites of KMTs appears to define the methylation state of the product; Tyr to Phe substitutions result in the formation of higher methylation states of lysine.6e The target lysine needs to be deprotonated for nucleophilic attack and an active site Tyr may also be responsible for deprotonation of the protonated lysine, although a water channel has also been suggested to play a role as a general base.^7^

Despite recent success in structural, mechanistic, and inhibition studies on KMTs,3a, 8 the biocatalytic potential of KMTs remains to be established.^9^ Enzymatic assays revealed that human KMTs exhibited a high degree of specificity for the methylation of lysine analogues that differed in stereochemistry, side‐chain length, and main chain.^10^ On the other hand, KMTs appear to have a limited ability to catalyze other alkylations of histones, including transfer of allyl, propargyl, and larger alkyl groups from AdoMet analogues bearing methyl group replacements.^11^ Herein, we report on investigations into KMT‐catalyzed ethylation of histone peptides that employ AdoEth and AdoSeEth cosubstrates (Figure 1 C).

## Results and Discussion

**Scheme 1:**
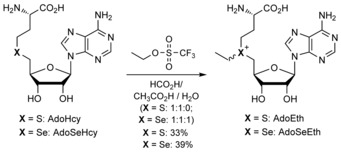
Chemical synthesis of AdoEth14a and AdoSeEth.

Analogues of AdoMet with methyl group replacements, including AdoEth and AdoSeEth, can be enzymatically synthesized from l‐methionine derivatives and adenosine triphosphate (ATP) using methionine adenosyltransferases (MATs) from different organisms.^12^ A pronounced product inhibition of the MAT enzymes, however, often limits the synthesis to small amounts with isolated enzymes.^13^ Larger amounts of cosubstrate analogues can be obtained by chemical synthesis (Scheme 1). AdoHcy^14^ or *Se*‐adenosylselenohomocysteine (AdoSeHcy)11a, 11b, 11h are typically reacted with alkylating agents under slightly acidic conditions with a mixture of formic and acetic acid. These conditions guide regioselective alkylation of the sulfur or selenium atom because all other nucleophilic positions are transiently protected by protonation. During the synthesis of the more reactive selenonium analogue AdoSeEth, we noticed many byproducts. Fortunately, the formation of these byproducts could be efficiently suppressed by adding water to the mixture of formic and acetic acid (Scheme 1).

Generally, alkylations of AdoHcy under acidic conditions lead to both diastereoisomers (epimers) at sulfur in almost equal amounts.14a In the case of AdoEth, the two epimers formed in a 45:55 ratio (*S*/*R*). Both epimers were separated by means of reversed‐phase HPLC and only the *S* epimer (corresponding to the biologically active *S* epimer of AdoMet) was used in this study. However, in the case of AdoSeEth, the separation of both epimers by reversed‐phase HPLC was not possible and AdoSeEth was used as an epimeric mixture.

**Figure 2:**
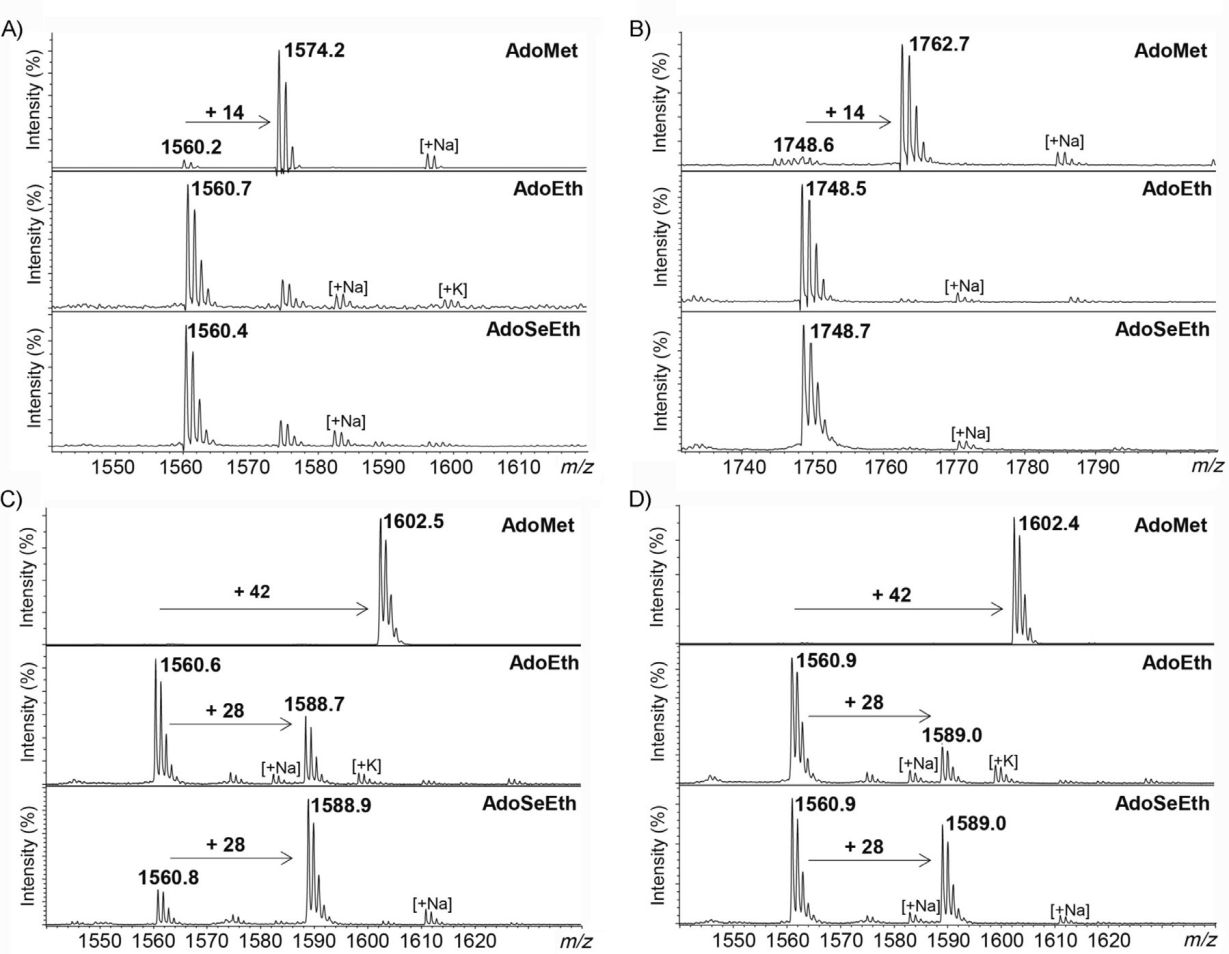
MALDI‐TOF MS assays showing methylation (top) and ethylation reactions (middle and bottom) of A) H3K4 by SETD7, B) H4K20 by SETD8, C) H3K9 by G9a, and D) H3K9 by GLP.

We then performed comparative enzymatic assays for KMT‐catalyzed methylation (with AdoMet) and ethylation (with AdoEth and AdoSeEth) of synthetic histone peptides by using MALDI‐TOF MS, as recently described;10a, 10b histone H3_1–15_ was used for studies with SETD7 (also known as KMT7), G9a (also known as KMT1C and EHMT2), and GLP (also known as KMT1D and EHMT1), and histone H4_13–27_ was used for studies with SETD8 (also known as KMT5A). MALDI‐TOF MS data confirmed that human KMTs catalyzed nearly quantitative methylation of histone peptides in the presence of AdoMet: H3K4me, H4K20me, H3K9me3, and H3K9me3 were formed in the presence of SETD7, SETD8, G9a, and GLP, respectively (Figure 2, top). Unlike monomethylation, SETD7 and SETD8 did not catalyze the ethylation of H3K4 and H4K20 with AdoEth or more reactive AdoSeEth within detection limits (Figure 2 A and B and Figures S1 and S2 in the Supporting Information). Human G9a and GLP, however, were able to catalyze the ethylation of H3K9; AdoSeEth was a superior ethylation agent to that of AdoEth (Figure 2 C and D). Although G9a and GLP catalyzed di‐ and trimethylation of H3K9, both enzymes were only able to catalyze monoethylation of H3K9; no di‐ and triethylation products were detected (Figure 2 C and D). A longer incubation time (5 h) with AdoEth led to the formation of 56 and 30 % of H3K9et by G9a and GLP, respectively (Figure S3), whereas almost complete (87 %) formation of H3K9et was observed after 5 h if AdoSeEth and G9a were used (Figure S4). Control experiments in the absence of AdoEth/AdoSeEth or G9a/GLP verified that ethylation reactions were catalyzed by KMT (Figures S5 and S6).

**Figure 3:**
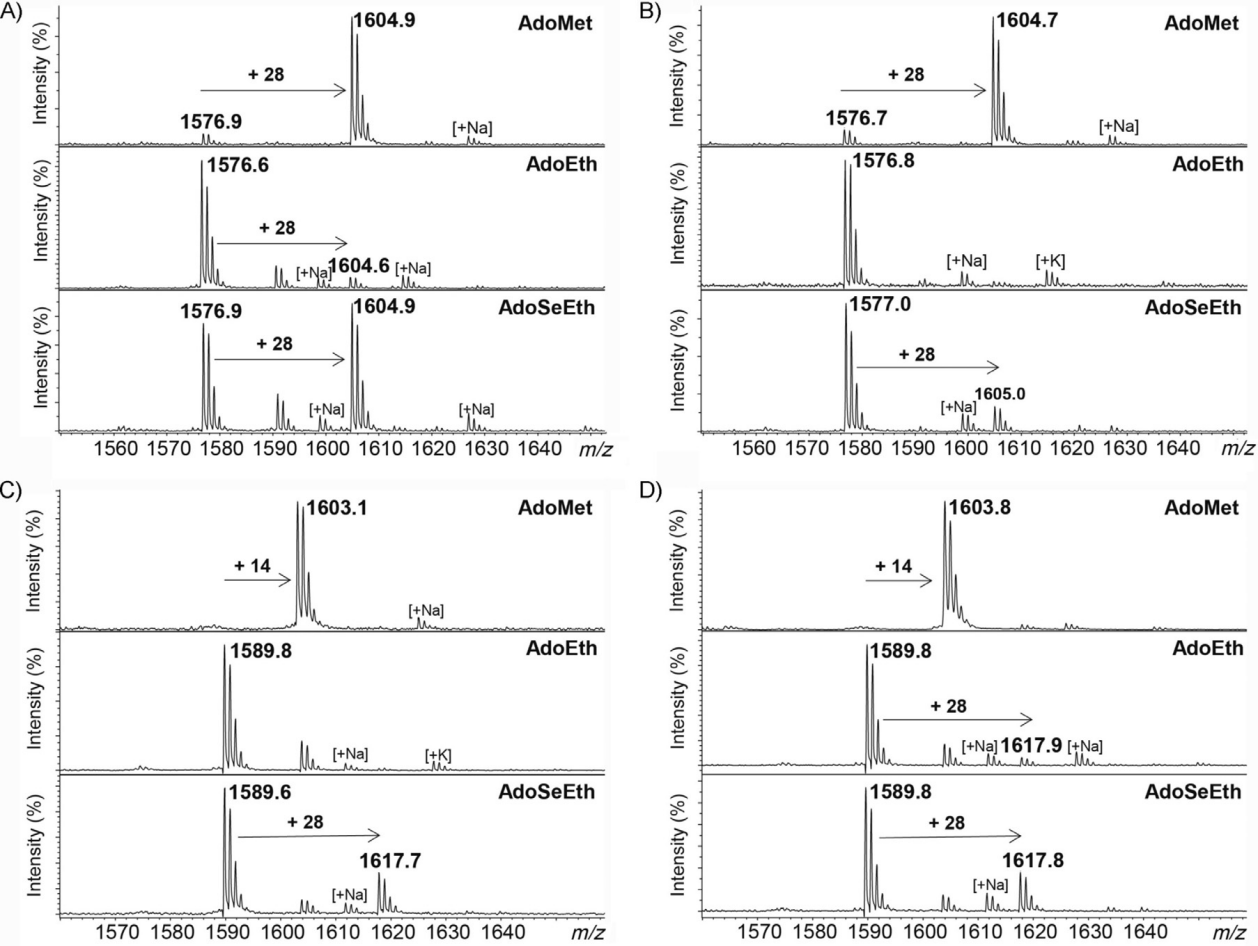
MALDI‐TOF MS assays showing methylation (top) and ethylation reactions (middle and bottom) of A) H3K9me by G9a, B) H3K9me by GLP, C) H3K9me2 by G9a, and D) H3K9me2 by GLP.

Having shown that G9a and GLP had the ability to catalyze monoethylation of H3K9, we next investigated potential enzymatic ethylation of biologically important methylated histones H3K9me and H3K9me2. G9a and GLP both poorly catalyzed ethylation of H3K9me (traces detected) in the presence of AdoEth, even upon prolonged incubation (Figures 3 A and B and S7). Both enzymes, however, produced detectable amounts of H3K9meet in the presence of more reactive AdoSeEth (Figures 3 A and B and S8). G9a, in particular, produced significant amounts (55 % after 3 h and 75 % after 5 h) of H3K9meet (Figures 3 A and B and S8). The observation that G9a and GLP have the capacity to catalyze the formation of H3K9meet is interesting because functionally related histone lysine demethylases PHF8, FBXL11, and JMJD2E were found to catalyze the removal of methyl and ethyl groups in H3K9meet; thus producing unmodified H3K9 (a substrate for G9a and GLP).^15^ Moreover, our MALDI‐TOF MS assays revealed that G9a and GLP also catalyzed the ethylation of H3K9me2, producing approximately 25 % of bulky H3K9me2et in the presence of AdoSeEth; only traces of H3K9me2et were observed if AdoEth was used as a cosubstrate (Figure 3 C and D). Prolonged incubation (5 h at 37 °C) led to increased amounts (41 %) of H3K9me2et in the presence of G9a and AdoSeEth, whereas AdoEth did not enhance the formation of the trialkylated product (Figures S9 and S10). Control experiments in the absence of G9a/GLP or AdoSeEth showed no ethylation of H3K9me and H3K9me2; thus implying that the reactions are catalyzed by KMT and that the ethyl groups in the H3K9meet and H3K9me2et products are derived from the AdoSeEth cosubstrate (Figures S11 and S12).

**Figure 4:**
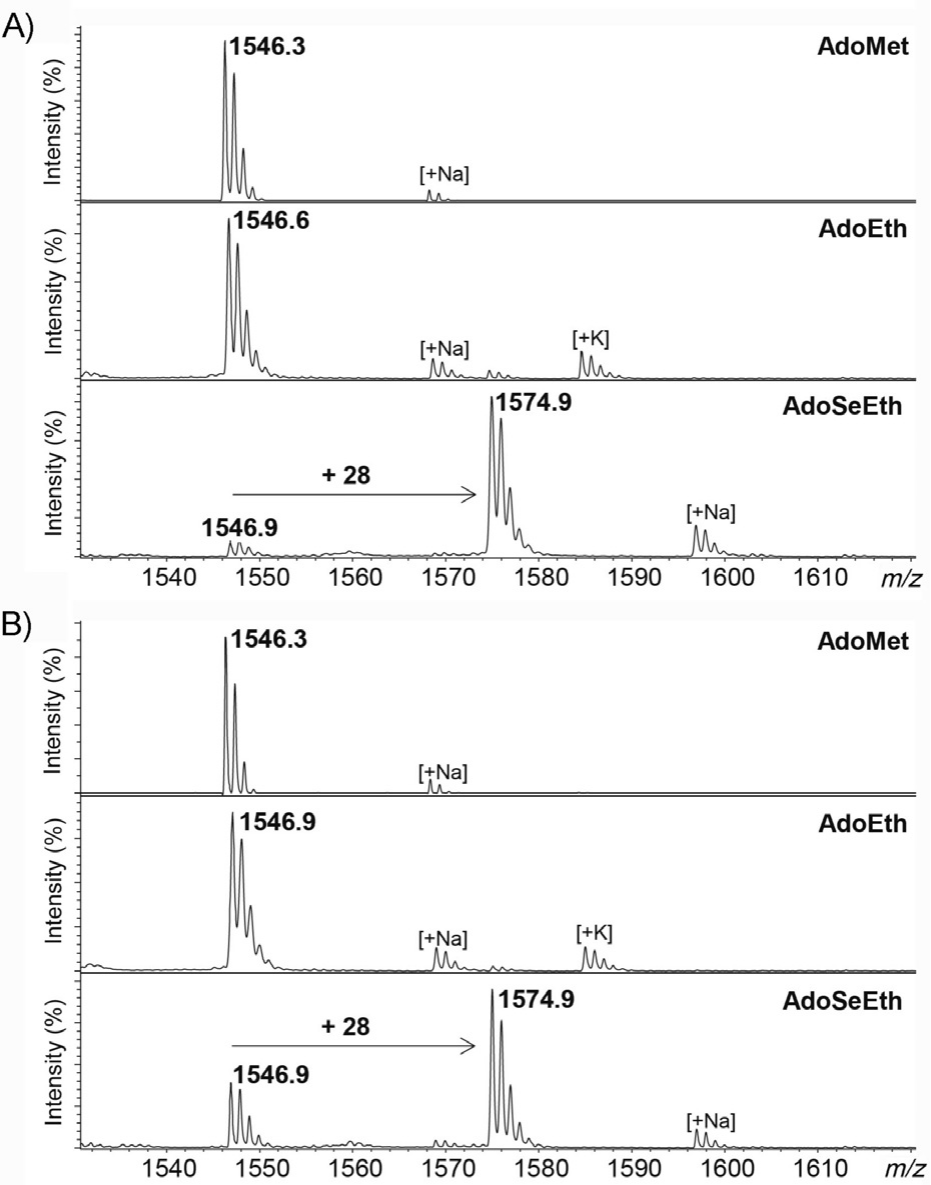
MALDI‐TOF MS assays showing methylation (top) and ethylation reactions (middle and bottom) of A) H3Orn9 by G9a and B) H3Orn9 by GLP.

Despite the fact that AdoSeEth and AdoEth can both act as ethylating agents in enzymatic assays, they do exhibit significant differences with respect to reactivity. In analogy with AdoMet/AdoSeMet,11b, 16 AdoSeEth appears to be more reactive, that is, a better alkylation agent than AdoEth. One notable difference between the two molecules is the bond length; the C−Se bond is longer (2.0 Å) than the C−S bond (1.8 Å), which makes the selenonium analogues more reactive. Due to the longer C−Se bond and higher reactivity of AdoSeEth, we hypothesized that KMTs might have the ability to catalyze the ethylation of ornithine, which is the lysine analogue shorter by one methylene group. In line with our earlier observation,10b we observed that G9a and GLP did not methylate H3Orn9, in the presence of AdoMet, within the limits of detection (Figure 4, top). Similarly, no G9a/GLP‐catalyzed ethylation of H3Orn9 was observed if AdoEth was used as a cosubstrate, even upon longer incubation times (Figures 4, middle, and S13). Interestingly, our MALDI‐TOF MS data showed that G9a and GLP predominantly catalyzed monoethylation of H3Orn9 in the presence of AdoSeEth; as in the case of H3K9, no diethylation of H3Orn9 was observed (Figure 4, bottom). A longer incubation time (5 h at 37 °C) led to nearly complete (96 %) and significant (78 %) formation of H3Orn9et with G9a and GLP, respectively (Figure S14). Controls without G9a/GLP or AdoSeEth again verified that ethylation reactions were catalyzed by KMT (Figure S15). We also examined potential G9a/GLP‐catalyzed ethylation of H3Dab9 peptide that possessed the lysine analogue 2,4‐diaminobutyric acid (Dab), which was shorter by two methylene groups. However, we did not detect any ethylated products in the presence of AdoEth and AdoSeEth, within detection limits (Figures S16 and S17). Finally, we also performed enzymatic assays with ornithine‐containing histone peptides H3Orn4 and H4Orn20 with human SETD7 and SETD8. Unlike monomethylation of H3K4, human SETD7 did not catalyze monoethylation of H3Orn4 in the presence of AdoEth or AdoSeEth (Figure S18). Similarly, despite high degrees of monomethylation of H4K20 by SETD8, the enzyme did not yield any H4Orn20et in the presence of AdoEth or AdoSeEth (Figure S19). This is in line with the absence of ethylation of H3K4 and H4K20 with AdoEth and AdoSeEth, as discussed above (Figure 2 A and B). Collectively, our enzymatic assays revealed that human G9a and GLP possessed the biocatalytic potential for ethylation of the shorter ornithine residue.

To obtain a better understanding of the G9a‐ and GLP‐catalyzed ethylation of H3K9 and H3Orn9, we performed kinetic experiments with different cosubstrate concentrations (Table 1 and Figures S20 and S21). However, G9a‐ and GLP‐catalyzed ethylation reactions with AdoEth were so slow that no multiple turnovers were obtained within the extended reaction time. Comparing the single turnover rate constant, *k*=0.046 min^−1^, of G9a in the presence of saturating AdoEth concentrations with *k*_cat_=11.6 min^−1^ obtained with AdoMet under multiple turnover (steady‐state) conditions indicates that alkylation with the natural cosubstrate is at least 200‐fold faster. Very similar kinetics results were obtained with GLP. Such a reduction in activity of two to three orders with AdoEth, compared with that of AdoMet, was also observed with DNA methyltransferases,14a and could be attributed to increased steric strain in the S_N_2‐type transition state for ethyl transfer, relative to that of methyl transfer. With AdoSeEth, the ethylation rate under saturating cosubstrate concentrations was about three to four times faster for both G9a and GLP; however, the true rate enhancement upon going from AdoEth to AdoSeEth might even be larger because AdoSeEth was employed as an epimeric mixture at the selenonium center (separation of the epimers by means of reversed‐phase HPLC was not possible, see above) and the epimer with the non‐natural *R* configuration might act as an inhibitor, as observed for (*R*)‐AdoMet.^17^ Furthermore, the ethylation rate of the side‐chain‐shortened substrate H3Orn9 with AdoSeEth is only reduced by 50 % compared with that of the H3K9 substrate with the natural target amino acid.

**Table 1:** Rate constants, *k*, for G9a*‐* and GLP‐catalyzed methylation and ethylation of H3K9 and H3Orn9 histone peptides with saturating AdoMet, AdoEth, and AdoSeEth concentrations. Reactions with AdoMet were obtained under steady‐state conditions, whereas reactions with AdoEth and AdoSeEth were too slow to reach steady state.

		AdoMet^[a]^	AdoEth^[b]^	AdoSeEth^[c]^	
		H3K9	H3K9	H3K9	H3Orn9
G9a	*k* [min^−1^]	11.6±0.61	0.046±0.005	0.21±0.01	0.12±0.01
GLP	*k* [min^−1^]	9.90±0.44	0.058±0.003	0.16±0.01	0.10±0.003

[a] No methylation was observed with H3Orn9 and AdoMet. [b] No ethylation was observed with H3Orn9 and AdoEth. [c] AdoSeEth was employed as an epimeric mixture at the selenonium center.

**Figure 5:**
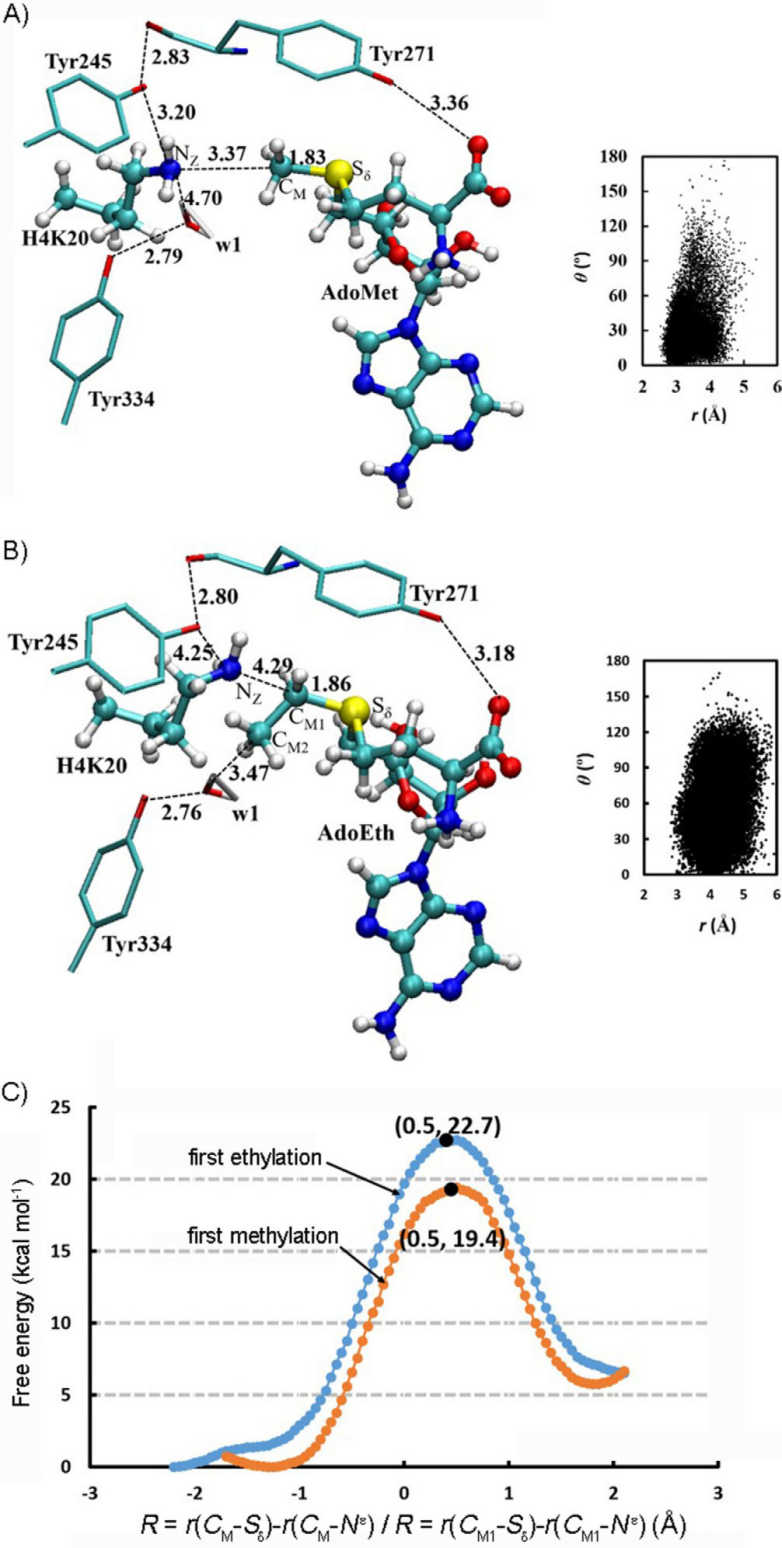
A) Representative active‐site structure of the reactant complex of SETD8 for the first methylation with AdoMet and lysine (H4K20) obtained from the QM/MM molecular dynamics (MD) simulations. The distribution map on the right shows the alignment of N^*ϵ*^H_2_ and the transferable methyl group in the reactant complex, in terms of the distance (*r*) between N^*ϵ*^ and C_M_ and the angle (*θ*) between the direction of the electron lone pair on N^*ϵ*^ and the C_M_−S bond. SETD8 is shown in sticks, and AdoMet and lysine are in balls and sticks. Some average distances from the simulations are also given [Å]. B) Representative active‐site structure of the reactant complex of SETD8 for the first ethylation with AdoEth and lysine (H4K20), along with the *r*(C_M1_⋅⋅⋅N^*ϵ*^) and *θ* distribution map obtained from QM/MM MD simulations. C) Free energy (potential of mean force) profiles for the methylation/ethylation reactions in SETD8, as a function of the reaction coordinate (*R*=*r*(C_M_−S_δ_)−*r*(C_M_−N^*ϵ*^)/*R*=*r*(C_M1_−S_δ_)−*r*(C_M1_−N^*ϵ*^)). The free energy profile for the first methylation step: orange line with a free energy barrier of 19.4 kcal mol^−1^ and the location of the transition state is at around 0.5. The free energy profile for the first ethylation step: blue line with a free energy barrier of 22.7 kcal mol^−1^.

We then performed quantum mechanics/molecular mechanics (QM/MM) investigations to rationalize experimental observations on the KMT‐catalyzed ethylation of lysine residues. Because the parameters for selenium are still not available in the semiempirical QM DFTB3 method, only simulations for the ethylation reactions involving AdoEth have been performed herein. The average active‐site structures of the reactant complexes for the first methylation and first ethylation in SETD8 are compared in Figure 5 (active‐site structures near the transition state are shown in Figure S22); the distribution maps of *r*(C_M_−N^ϵ^)/*r*(C_M1_−N^ϵ^) and *θ* are also given in each case. As observed, the alignment of the electron lone pair on N^ϵ^ of the target lysine with transferable ethyl groups (Figure 5 B) is significantly worse than that with the transferable methyl group (Figure 5 A). For instance, the average distance between N^ϵ^ of lysine and C_M1_ is 4.3 Å in Figure 5 B, whereas it is 3.4 Å between N^ϵ^ and C_M_ in Figure 5 A. The poor alignment between the ethyl donor and acceptor positions is also reflected in the distribution map. The free energy profiles for the first methylation and first ethylation reactions in SETD8 are given in Figure 5 C. Consistent with the structures of the reactant complexes, the free energy barrier for the ethylation reaction (22.7 kcal mol^−1^) is considerably higher than that of methylation (19.4 kcal mol^−1^). The simulation results indicate that, although SETD8 can catalyze the monomethylation of H4K20, it may not be able to do so for the ethylation reaction; this is consistent with the experimental data (Figure 2 B).

**Figure 6:**
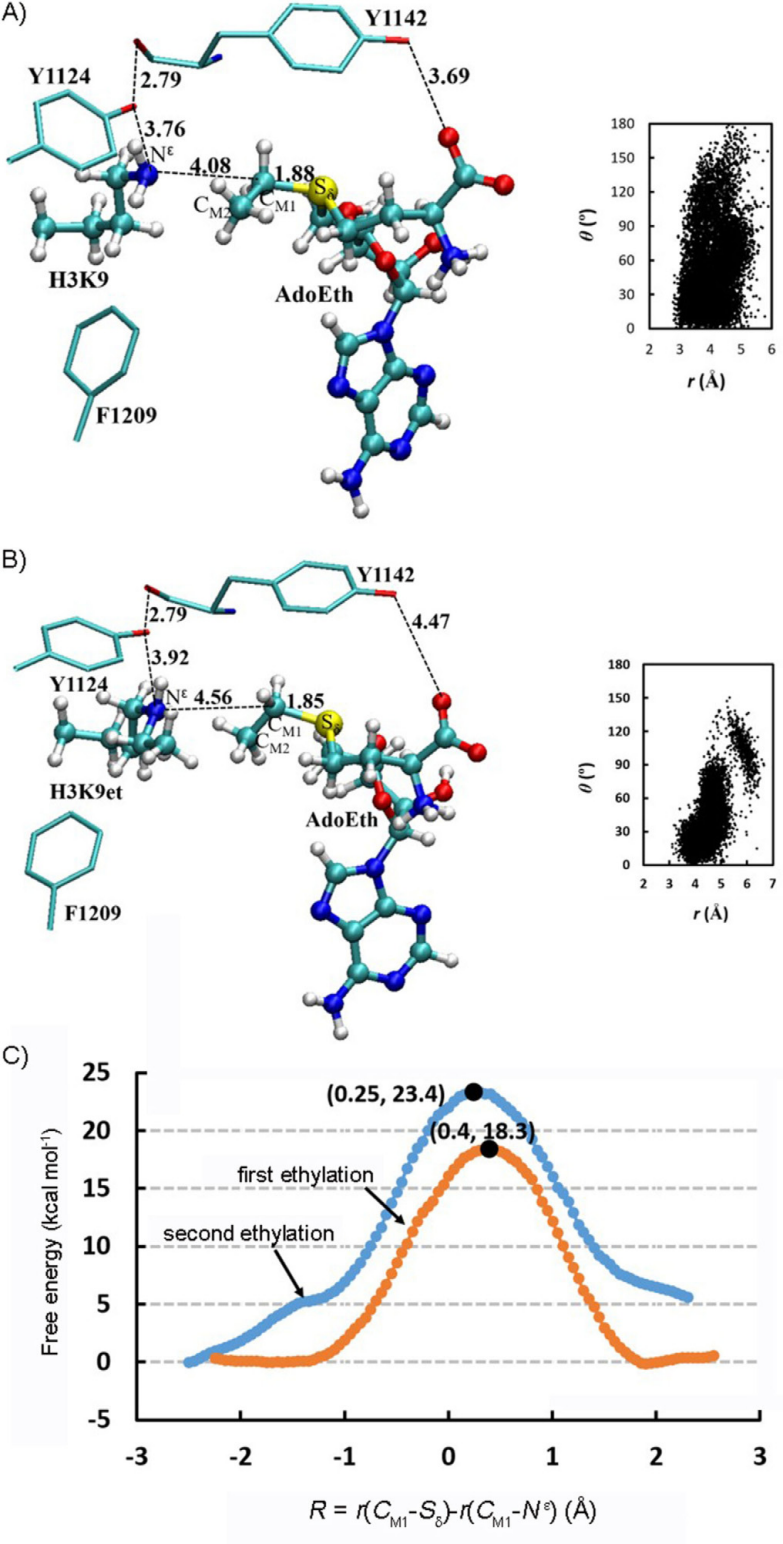
A) Average structure obtained from MD simulations for the first ethyl transfer from AdoEth to H3K9 in GLP, along with the distribution map during the MD simulations. Some average distances of potential hydrogen bonds between AdoEth/substrate and nearby residues are shown [Å]. B) Average structure obtained from MD simulations for the second ethyl transfer from AdoEth to H3K9et in GLP. C) Free energy changes for mono‐ and diethylation of H3K9 in GLP. Monoethylation: orange line with a free energy barrier of 18.3 kcal mol^−1^. Diethylation: blue line with a free energy barrier of 23.4 kcal mol^−1^.

The average active‐site structures of the reactant complexes for the first and second ethyl transfers in GLP are given in Figure 6 A and B, respectively (active‐site structures near the transition state are shown in Figure S23). Figure 6 A and B shows that the alignment of the electron lone pair on N^ϵ^ of the target lysine with the transferable ethyl group in GLP is significantly better for the first ethyl transfer step (with a shorter average C_M1_⋅⋅⋅N^*ϵ*^ distance of 4.08 Å and higher population of near attack conformations) compared with that for the second ethyl transfer step (with an average C_M1_⋅⋅⋅N^*ϵ*^ distance of 4.56 Å). This is in contrast with cases for the first and second methyl transfers in GLP in which the target lysine and methyllysine can be well aligned, respectively, with the transferable methyl group (Figure S24). The free energy profiles for the first and second ethylation reactions in Figure 6 C demonstrate that, although GLP may catalyze the first ethylation reaction (with a free energy barrier of 18.3 kcal mol^−1^, which is higher than that of 17.0 kcal mol^−1^ for the first methylation reaction10b), it is unlikely to be able to catalyze the second ethylation reaction with AdoEth because the free energy barrier becomes significantly higher (23.4 kcal mol^−1^). These results are in agreement with the observed monoethylated product, H3K9et, and lack of the diethylated product H3K9et2 in MALDI‐TOF assays (Figure 2 D).

## Conclusion

We have demonstrated that human KMTs G9a and GLP have the capacity to catalyze monoethylation of H3K9 in the presence of AdoEth and AdoSeEth cosubstrates. Enzymatic assays revealed that AdoSeEth was a superior ethylating agent to AdoEth, but comparatively poorer cosubstrate than that of natural AdoMet. The ability of AdoEth to act as an ethylating agent for histone ethylation in cells^18^ and in vitro, as investigated herein, might have some biological relevance because its precursor, ethionine, is a toxic compound that can be converted into AdoEth by eukaryotic MAT enzymes.^19^ Computational work revealed that the molecular origin for more efficient enzymatic methylation over that of ethylation of lysine residues in histones lay in more optimal alignment of the smaller methyl group of AdoMet, relative to that of the larger ethyl group of AdoEth. Our examinations also revealed that G9a and GLP catalyzed the ethylation of histone H3, bearing biologically relevant methylated lysine residues (H3K9me and H3K9me2), and histone H3 peptide possessing ornithine (H3Orn9), which is shorter by one methylene group, in the presence of AdoSeEth. Collectively, this work highlights the biocatalytic potential of selected human KMTs and expands the substrate scope for KMT‐catalyzed alkylation of histones. It is envisioned that this work, along with recent investigations into KMT‐catalyzed alkylation of proteins, including histones, will advance our basic understanding of KMT catalysis.

## Experimental Section

**Materials**: AdoSeHcy11h and AdoEth14a were prepared as described previously.

**Synthesis**

**Ethyl triflate**: Ethyl triflate was obtained by following a slightly modified literature procedure.^20^ Polyvinylpyridine (1.21 g) was suspended in dichloromethane (27 mL) cooled on an ice bath. Trifluoromethanesulfonic anhydride (1.55 g, 5.49 mmol) was added dropwise, and then ethanol (240 mg, 5.21 mmol) in dichloromethane (2.75 mL) was added within 2 min. The ice bath was removed and stirring was continued for another 10 min at room temperature. The solid was filtered off, and the solvent was removed under reduced pressure to yield a slightly brown liquid (754 mg, 4.23 mmol, 81 %). ^1^H NMR (300 MHz, CDCl_3_): *δ*=1.45 (t, *J=*7.0 Hz, 3 H; H2), 4.55 ppm (q, *J=*7.2 Hz, 2 H; H1).

**AdoSeEth**: AdoSeHcy (20 mg, 46.4 μmol) was dissolved in a 1:1 mixture of formic and acetic acid (1.95 mL) and water (0.98 mL) was added. The solution was supplemented with ethyl triflate (1.49 g, 8.37 mmol), and the reaction mixture was stirred at room temperature for 1 h. The reaction was supplemented with water (6 mL), and the aqueous phase was extracted three times with diethyl ether (7.5 mL each time). Purification of the product in the aqueous phase was performed by means of preparative reversed‐phase HPLC (Prontosil‐ODS 5 μm, 120 Å, 250×20 mm, Bischoff, Leonberg, Germany). Compounds were eluted with methanol (linear gradients from 0 to 7.8 % in 15 min and to 78 % in 5 min) in aqueous trifluoroacetic acid (0.01 %) and a flow of 10 mL min^−1^. Compounds were detected at *λ*=260 and 272 nm. The two epimers (at selenium) both eluted with a retention time of around 9.9 min and could not be separated. Product‐containing fractions were collected and the solvents were removed by lyophilization. The remaining solid was dissolved in water (1.5 mL) and the yield (8.32 mg, 18.1 μmol, 39 %) was determined by UV spectroscopy (*ϵ* ^260^=15 400 L mol^−1^ cm^−1^). ESI‐MS: *m*/*z* (%): 461.1 (100) [*M*]^+^, 360.2 (8) [5′‐ethylseleno‐5′‐deoxyadenosine+H]^+^, 250.4 (17) [*M*−ethionine]^+^, 136.2 (9) [adenine+H]^+^.

**Expression and purification of KMTs**: The four wild‐type human KMTs (SETD7, SETD8, G9a, and GLP) were expressed and purified as described previously.10a, 10b The wild‐type sequences of human methyltransferases were as follows: SETD8 (aa 186–352), SETD7 (aa 1–366), G9a (aa 913–1193), and GLP (aa 951–1235). Briefly, overnight cultures of *Escherichia coli* Rosetta BL21 (DE3)pLysS harboring expression plasmids were grown at 37 °C with shaking for 18 h in lysogeny broth (LB) medium supplemented with kanamycin and chloramphenicol. Expression was induced at OD_600_ (approximately 0.5–0.6) by adding isopropyl‐β‐d‐thiogalactopyranoside (IPTG) and shaking continued at 16 °C for 20 h. Cells were harvested by centrifugation at 4 °C for 15 min, and the cell pellets were resuspended in lysis buffer. Cells were lysed by using a Soniprep 150 sonicator for 20 s (8×) with 90 s intervals, keeping the cells chilled in an ice water bath at all times. After centrifugation, the supernatant was loaded onto Ni‐charged His‐tag binding resin equilibrated with column buffer. Resins were washed thoroughly with column buffer, followed by washing buffer, and protein was eluted with elution buffer under a linear gradient concentration of imidazole. The protein was then applied to size exclusion chromatography (SEC) by using a Superdex 75 column (GE Healthcare). Purified proteins were concentrated by employing Amicon ultra centrifugal filter units (Millipore) with suitable molecular weight cutoffs (10 kDa). Protein concentration was determined by employing a Nanodrop DeNovix DS‐11 spectrophotometer and the purity was monitored by means of SDS‐PAGE on a 4–15 % gradient polyacrylamide gel (Bio‐Rad). Enzymes were aliquoted and stored at −80 °C for future use.

**Enzymatic assays by means of MALDI‐TOF MS**: The reactions were performed in a total volume of 25 μL in an Eppendorf vial by using a thermomixer. A typical enzymatic assay included histone peptides (40 μm), cosubstrate AdoMet (200 μm with SETD8 and SETD7; 500 μm with GLP and G9a), AdoEth (1 mm) or AdoSeEth (1 mm), and KMT enzyme (2 μm) in a reaction buffer of 50 mm Tris**⋅**HCl at pH 8.0. The reactions were incubated at 37 °C and aliquots were removed from the reaction vial at different time points (1, 3, and 5 h) to measure the conversion of histone peptide substrates into alkylated products. The reaction was stopped by mixing the reaction mixture (3 μL) with MeOH (3 μL). An aliquot (3 μL) of these samples was directly mixed onto the MALDI target plate with α‐cyano‐4‐hydroxycinnamic acid (CHCA) matrix (3 μL, 5 mg mL^−1^ in 50 % (*v*/*v*) acetonitrile/water) and dried in air. Peptide substrate masses were measured in positive‐ion reflector mode. Full mass scans were acquired in the *m*/*z* range of 500–4000. Each mass spectrum was generated from data derived from 3–5 single laser shots in 200 shot steps from different positions of the sample spot. All spectra were manually acquired by using a Microflex mass spectrometer and FlexControl software, and the data were annotated by employing FlexAnalysis software (Bruker Daltonics, Germany). The following methylation and ethylation species were observed: mono‐ (+14 Da), di‐ (+28 Da), and trimethylation (+42 Da), and monoethylation (+28 Da). All methylation and ethylation experiments were performed in replicate.

**Enzyme kinetics analyses**: The kinetics parameters (*k*, *K*) were determined by incubating G9a or GLP (2 μm for AdoEth; 1 μm for AdoSeEth) and histone peptide (25 μm 15‐mer H3K9 or H3Orn9) in 50 mm Tris**⋅**HCl, pH 8.0, in the presence of various concentrations of AdoEth (0–250 μm) or AdoSeEth (0–250 μm). The reactions (final volume=20 μL) were incubated at 37 °C (700 rpm) for 15 min, after which they were quenched by adding an equal volume of MeOH. For analysis by MALDI‐TOF MS, the quenched reaction mixture (2 μL) was mixed with a saturated solution of CHCA (6 μL; 1:1 (*v*/*v*) in MeCN/double‐distilled H_2_O+0.1 % trifluoroacetic acid). From this, 1 μL was spotted onto the MALDI plate for crystallization. The enzymatic activity was determined by using the peak areas (including all isotopes) for each alkylation state. To obtain the kinetic parameters, data were plotted and fitted to the nonlinear regression enzyme kinetics function *k*_cat_ by using GraphPad PRISM software (Figure S20). The kinetics profiles for AdoMet (Figure S21) were obtained by incubation of G9a or GLP (50 nm), histone peptide (10 μm 15‐mer H3K9), and AdoMet (0–15 μm) in 50 mm Tris**⋅**HCl, pH 8.0, buffer for 3 min at 37 °C (700 rpm). MALDI‐TOF MS measurements and data analysis was performed in the same way as that for AdoEth and AdoSeEth described above.

**QM/MM studies**: QM/MM free energy (potential of mean force) and MD simulations were performed to study the active‐site dynamics of SETD8 and GLP and to calculate the free energy profiles of ethyl transfers from AdoEth to lysine and its ethylated form by using the CHARMM program.^21^ The −CH_2_−CH_2_−S^+^(Et)−CH_2_− part of AdoEth and lysine/ethyllysine chain were treated by QM and the rest of the system by MM. The link‐atom approach^22^ was applied to separate the QM and MM regions. A modified TIP3P water model^23^ was employed for the solvent, and the stochastic boundary MD method^24^ was used for the QM/MM simulations. The system was separated into a reaction zone and a reservoir region, and the reaction zone was further divided into a reaction region and a buffer region. The reaction region was a sphere with radius, *r*, of 20 Å, and the buffer region extended over 20 Å≤*r*≤22 Å. The reference center for partitioning the system was chosen to be the *N* ^*ϵ*^ atom of the target lysine. The resulting systems contained around 5800 atoms for GLP (or 5400 atoms for SETD8), including about 700–900 water molecules. The DFTB3 method^24^, ^25^ implemented in CHARMM was used for the QM atoms. The semiempirical approach adopted herein was used previously on a number of systems, and the results seemed to be reasonable.^26^ The all‐hydrogen CHARMM potential function (PARAM27)^27^ was used for the MM atoms.

The initial coordinates for the reactant complexes of methylation/ethylation were based on the crystallographic complexes (PDB IDs: 2BQZ and 3HNA for SETD8 and GLP, respectively); a methyl/ethyl group was manually added to SAH to change it to AdoMet/AdoEth and the methyl group on methyllysine was manually deleted to generate the target lysine. The initial structures for the entire stochastic boundary systems were optimized by using the steepest descent (SD) and adopted‐basis Newton–Raphson (ABNR) methods. The systems were gradually heated from 50.0 to 298.15 K in 50 ps. A 1 fs time step was used for integration of the equation of motion, and the coordinates were saved every 50 fs for analyses. The 1.5 ns QM/MM MD simulations were performed for each of the reactant complexes, and similar approaches have been used previously.7a, 28

The umbrella sampling method^29^ implemented in the CHARMM program, along with the weighted histogram analysis method (WHAM),^30^ was applied to determine changes of the free energy (potential of mean force, PMF) as a function of the reaction coordinate for methyl/ethyl transfer from AdoMet/AdoEth to the target lysine/ethyllysine in SETD8 and in GLP, respectively. The reaction coordinate was defined as a linear combination of *r*(C_M_−S_δ_) and *r*(C_M_−N^*ϵ*^) for methylation (*R*=*r*(C_M_−S_δ_)−*r*(C_M_−N^*ϵ*^)) or *r*(C_M1_−N^*ϵ*^) and *r*(C_M1_−S_δ_) for ethylation (*R*=*r*(C_M1_−S_δ_)−*r*(C_M1_−N^*ϵ*^); see Figure 5 for the atom designation). Thirty windows were used and 50 ps production runs were performed for each window after 50 ps equilibration. The force constants of the harmonic biasing potentials used in the PMF simulations were 50–400 kcal mol^−1^ Å^−2^.

## Conflict of interest

*The authors declare no conflict of interest*.

## Supporting information

As a service to our authors and readers, this journal provides supporting information supplied by the authors. Such materials are peer reviewed and may be re‐organized for online delivery, but are not copy‐edited or typeset. Technical support issues arising from supporting information (other than missing files) should be addressed to the authors.

Supplementary
