# Supplementary material for: Lysine Ethylation by Histone Lysine Methyltransferases
Source: Chembiochem. 2019 Oct 24;21(3):392–400. doi: 10.1002/cbic.201900359 (PMC7064923; doi:10.1002/cbic.201900359)
Supplement: Supplementary file 1 — Supplementary [file CBIC-21-392-s001.pdf]

CHEMBIOCHEM

Supporting Information

# Lysine Ethylation by Histone Lysine Methyltransferases

Abbas H. K. Al Temimi,[a] Michael Martin,[b] Qingxi Meng,[c] Danny C. Lenstra,[a] Ping Qian,[c] Hong Guo,*[d, e] Elmar Weinhold,*[b] and Jasmin Mecinović*[a, f]

cbic_201900359_sm_miscellaneous_information.pdf

# Table of Contents

1. MALDI-TOF MS supplementary figures S2

2. Enzyme kinetics analyses S21

3. Computational figures S23

## 1. MALDI-TOF MS supplementary figures

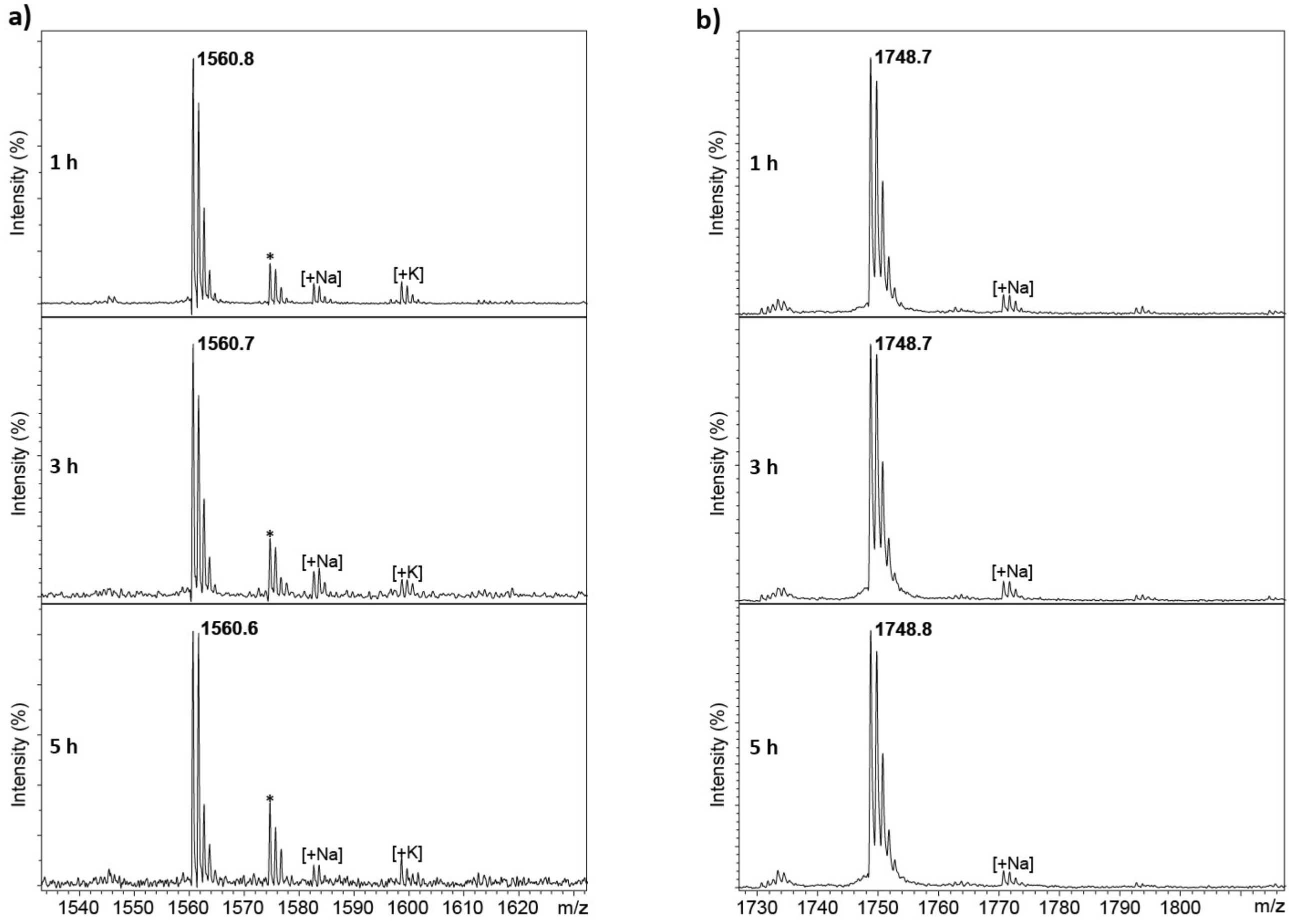


**Figure S1**. MALDI-TOF MS analysis showing a lack of ethylation for (**a**) H3K4 (40 μM) in the presence of SETD7 (2 μM) and AdoEth (1 mM) after incubation for 1 h (top panel), 3 h (middle panel), and 5 h (bottom panel) at 37 °C; (**b**) H4K20 (40 μM) in the presence of SETD8 (2 μM) and AdoEth (1 mM) after incubation for 1 h (top panel), 3 h (middle panel), and 5 h (bottom panel) at 37 °C. * The H3K4me signal derives from the presence of residual AdoMet bound to SETD7 during expression and purification.

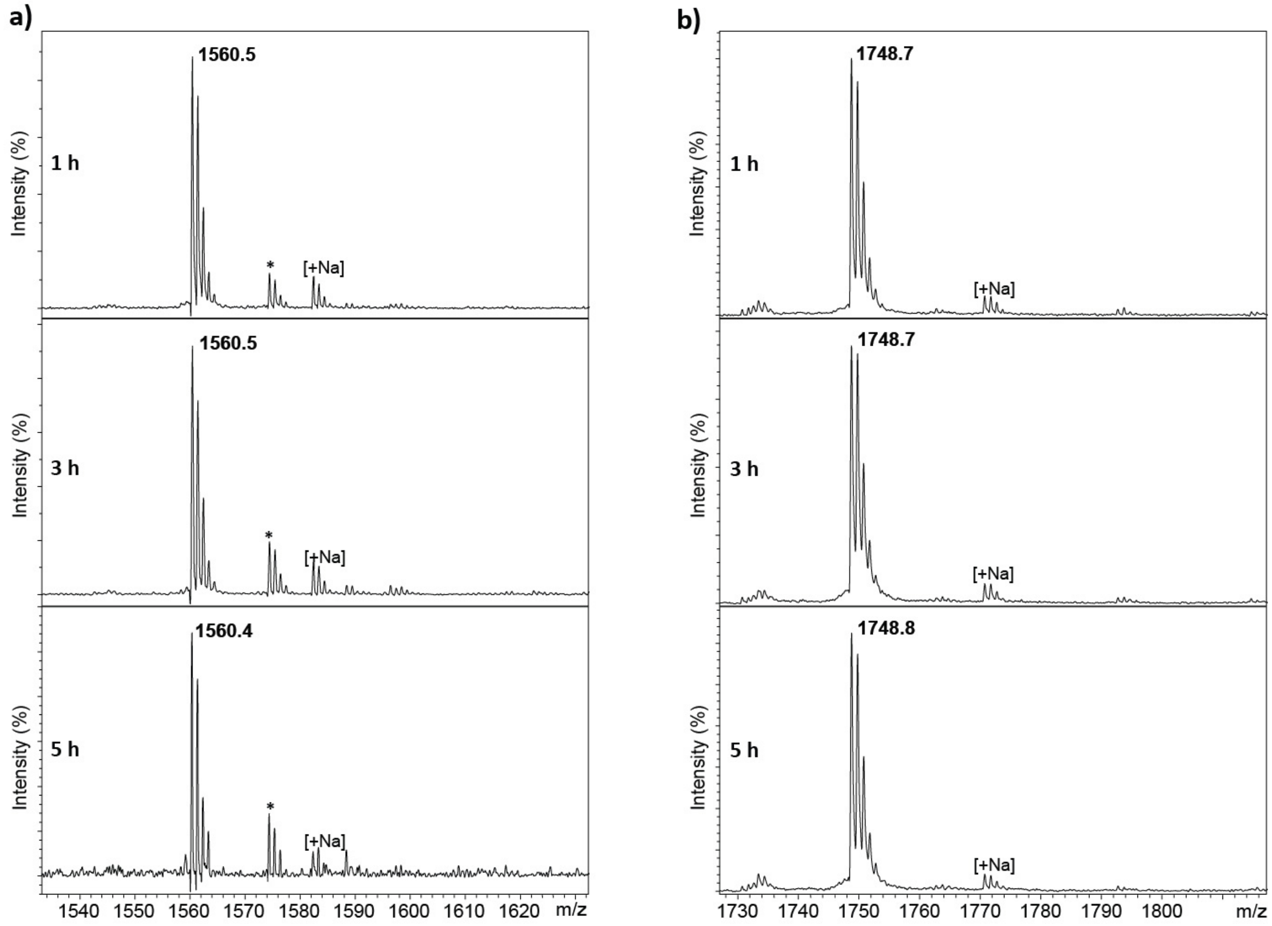


**Figure S2**. MALDI-TOF MS analysis showing a lack of ethylation for (**a**) H3K4 (40 µM) in the presence of SETD7 (2 µM) and AdoSeEth (1 mM) after incubation for 1 h (top panel), 3 h (middle panel), and 5 h (bottom panel) at 37 °C; (**b**) H4K20 (40 µM) in the presence of SETD8 (2 µM) and AdoSeEth (1 mM) after incubation for 1 h (top panel), 3 h (middle panel), and 5 h (bottom panel) at 37 °C. * The H3K4me signal derives from the presence of residual AdoMet bound to SETD7 during expression and purification.

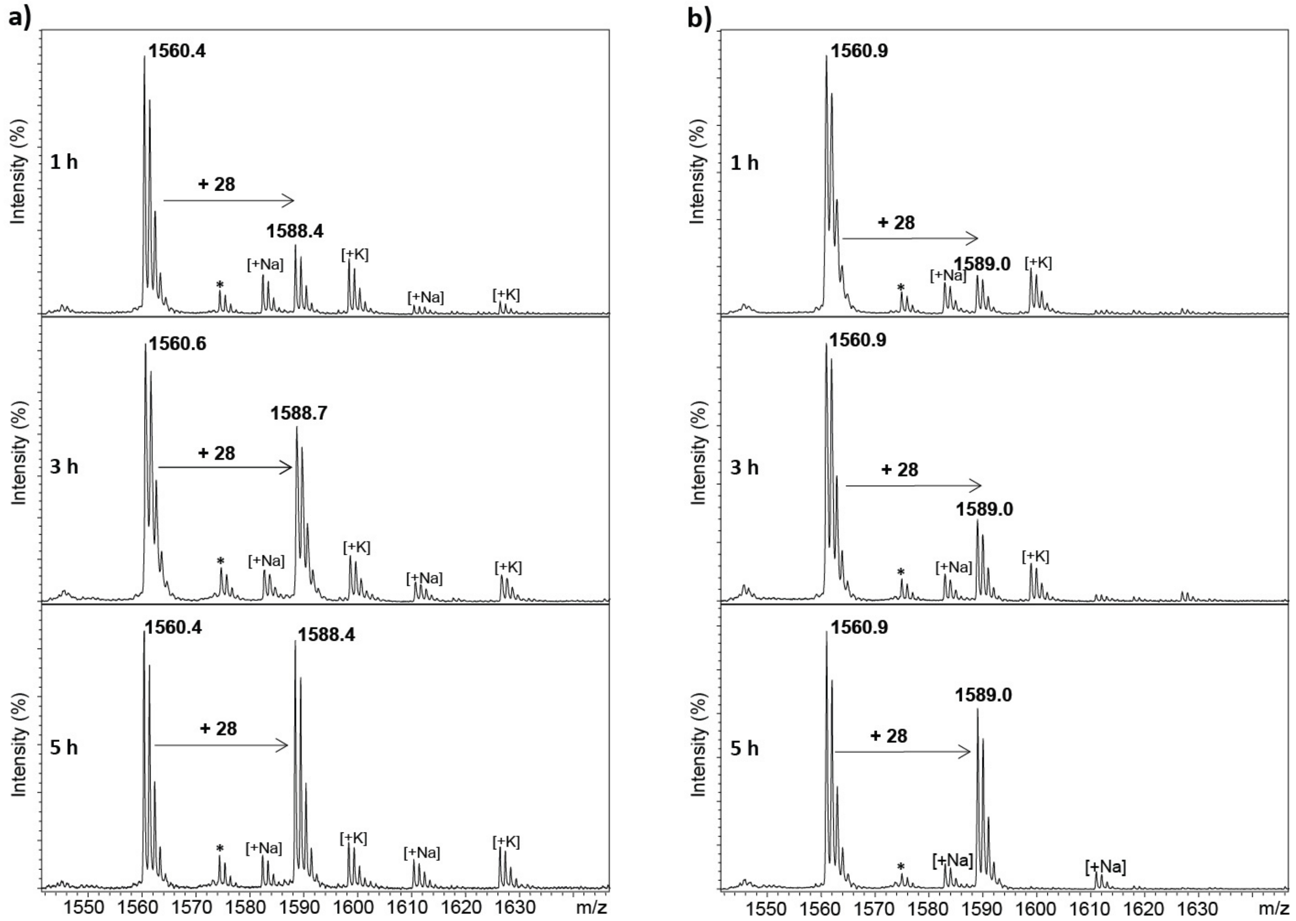


**Figure S3**. MALDI-TOF MS data showing (**a**) G9a (2 µM) catalyzed ethylation of H3K9 (40 µM) in the presence of AdoEth (1 mM) after incubation for 1 h (top panel), 3 h (middle panel), 5 h (bottom panel) at 37 °C; (**b**) ) GLP (2 µM) catalyzed ethylation of H3K9 (40 µM) in the presence of AdoEth (1 mM) after incubation for 1 h (top panel), 3 h (middle panel), 5 h (bottom panel) at 37 °C. * The H3K9me signal derives from the presence of residual AdoMet bound to G9a and GLP during expression and purification.

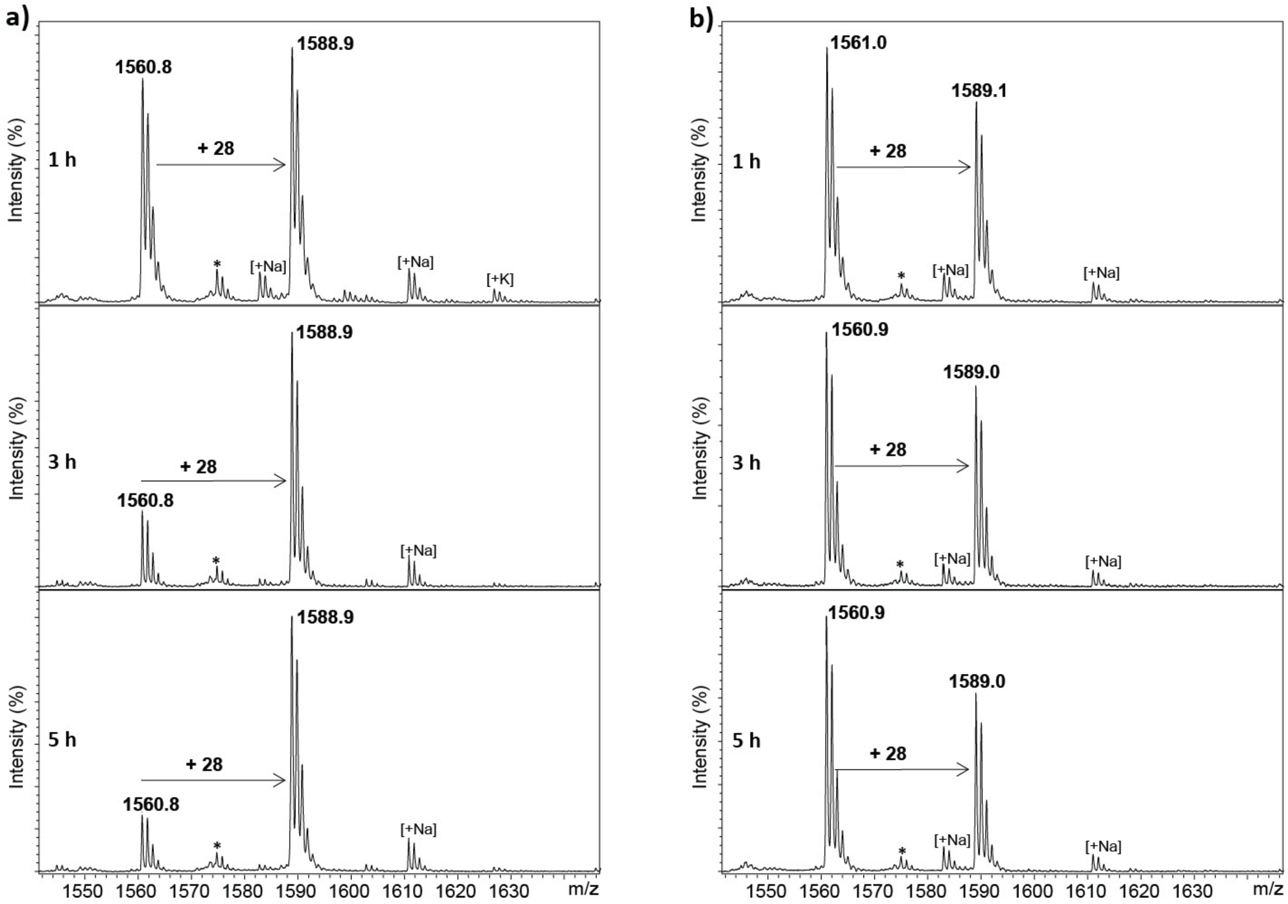


**Figure S4**. MALDI-TOF MS data showing (**a**) G9a (2 μM) catalyzed ethylation of H3K9 (40 μM) in the presence of AdoSeEth (1 mM) after incubation for 1 h (top panel), 3 h (middle panel), 5 h (bottom panel) at 37 °C; (**b**) GLP (2 μM) catalyzed ethylation of H3K9 (40 μM) in the presence of AdoSeEth (1 mM) after incubation for 1 h (top panel), 3 h (middle panel), 5 h (bottom panel) at 37 °C. * The H3K9me signal derives from the presence of residual AdoMet bound to G9a and GLP during expression and purification.

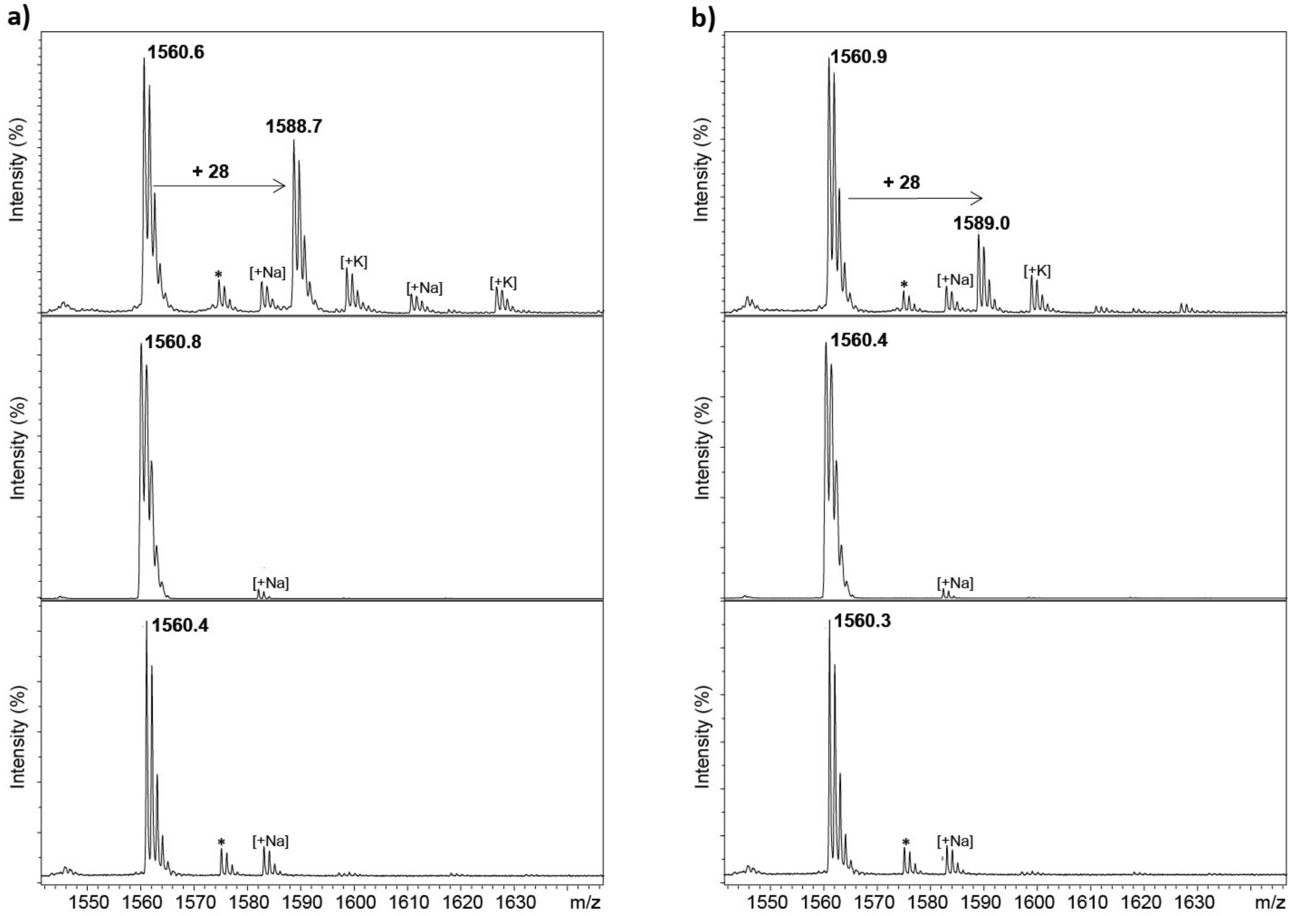


**Figure S5.** MALDI-TOF MS data showing (**a**) G9a (2 µM) catalyzed ethylation of H3K9 (40 µM) in the presence of AdoEth (1 mM) after 3 h (top panel) at 37 °C. Control reaction in the absence of G9a (middle panel). Control reaction in the absence of AdoEth (bottom panel). (**b**) GLP (2 µM) catalyzed ethylation of H3K9 (40 µM) in the presence of AdoEth (1 mM) after 3 h (top panel) at 37 °C. Control reaction in the absence of GLP (middle panel). Control reaction in the absence of AdoEth (bottom panel). * The H3K9me signal derives from the presence of residual AdoMet bound to G9a and GLP during expression and purification.

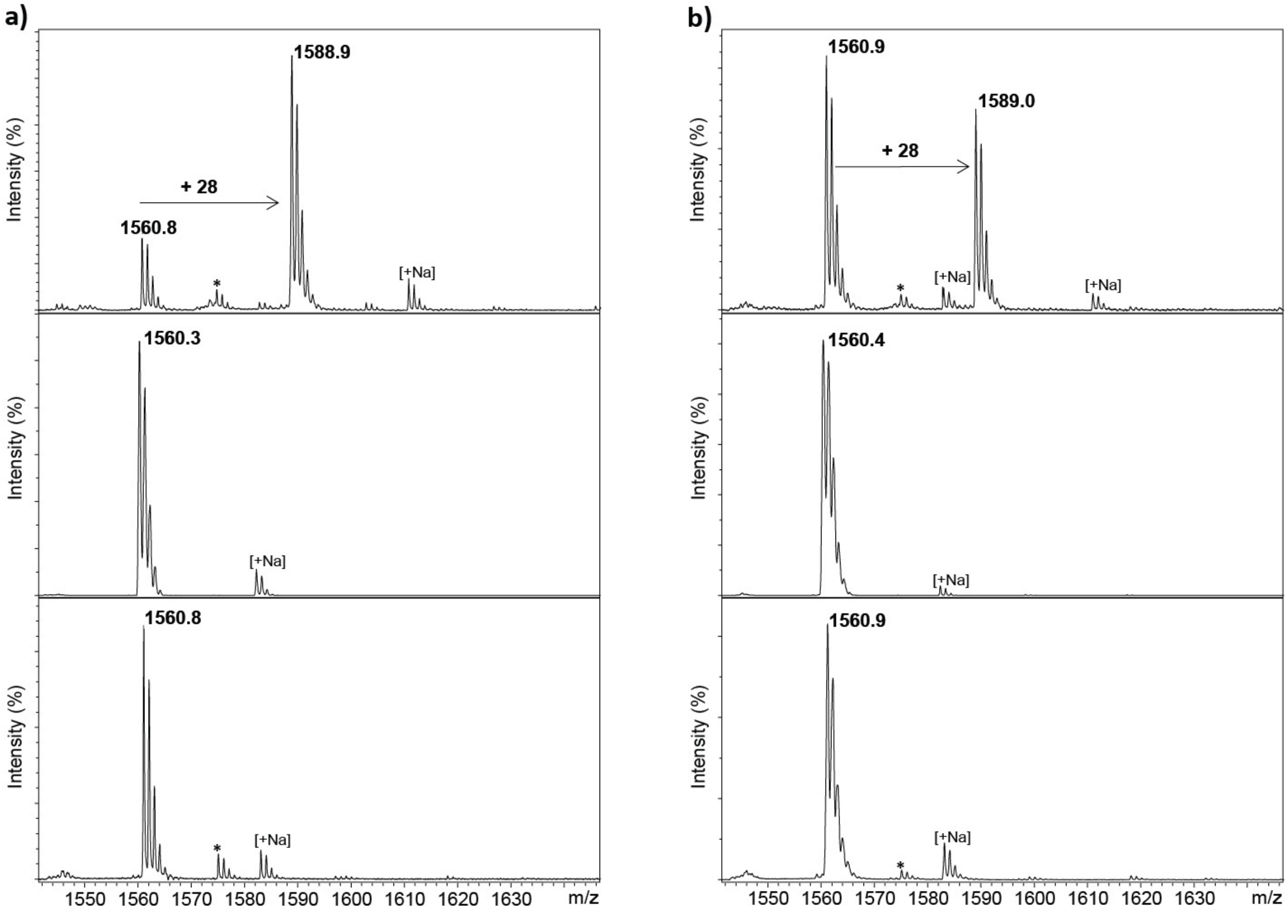


**Figure S6.** MALDI-TOF MS data showing (**a**) G9a (2 μM) catalyzed ethylation of H3K9 (40 μM) in the presence of AdoSeEth (1 mM) after 3 h (top panel) at 37 °C. Control reaction in the absence of G9a (middle panel). Control reaction in the absence of AdoSeEth (bottom panel). (**b**) GLP (2 μM) catalyzed ethylation of H3K9 (40 μM) in the presence of AdoSeEth (1 mM) after 3 h (top panel) at 37 °C. Control reaction in the absence of GLP (middle panel). Control reaction in the absence of AdoSeEth (bottom panel). * The H3K9me signal derives from the presence of residual AdoMet bound to G9a and GLP during expression and purification.

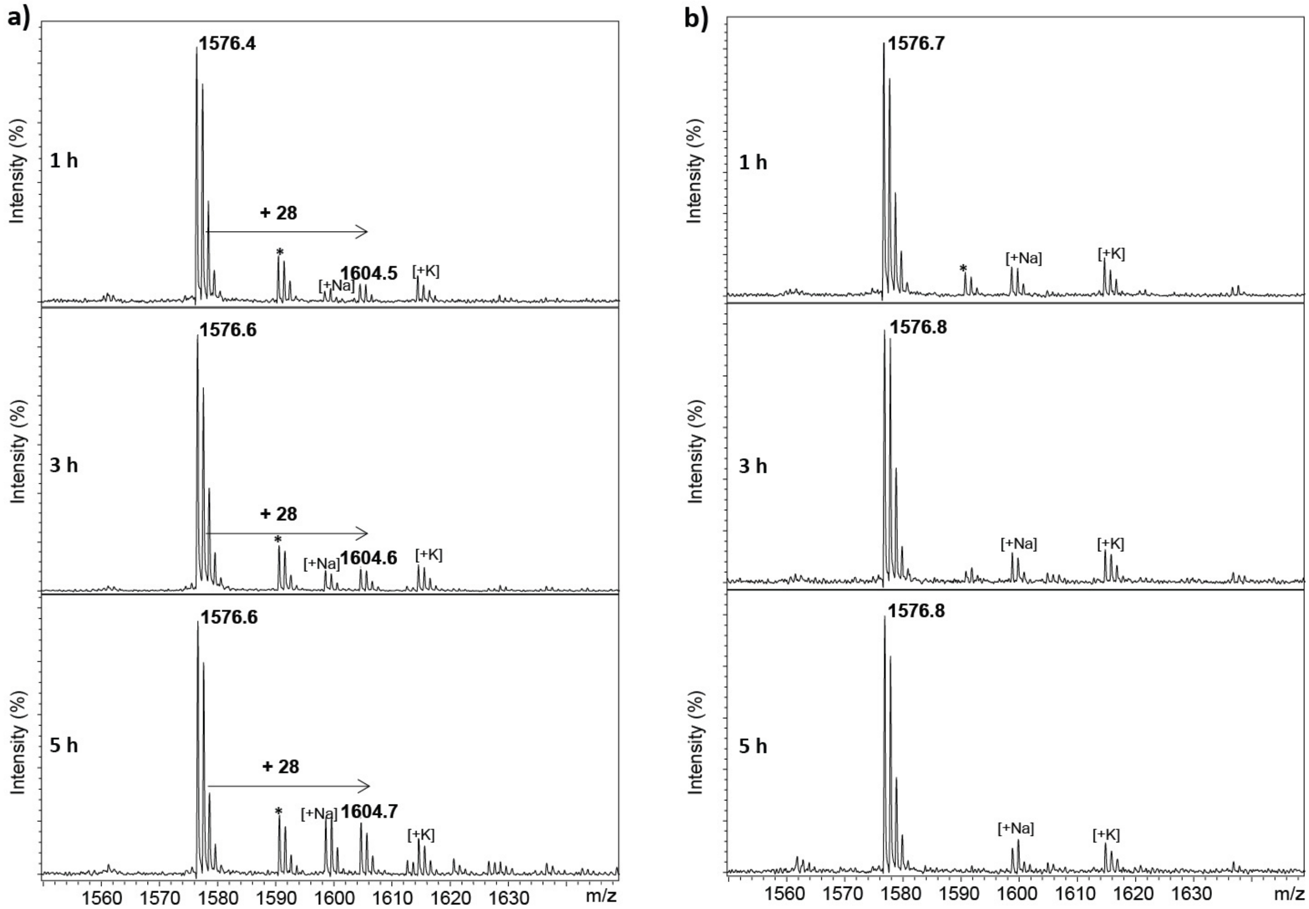


**Figure S7**. MALDI-TOF MS data showing (**a**) G9a (2 µM) catalyzed traces amount of ethylation of H3K9me (40 µM) in the presence of AdoEth (1 mM) after incubation for 1 h (top panel), 3 h (middle panel), 5 h (bottom panel) at 37 °C; (**b**) a lack of ethylation for H3K9me (40 µM) in the presence of GLP (2 µM) and AdoEth (1 mM) after incubation for 1 h (top panel), 3 h (middle panel), and 5 h (bottom panel) at 37 °C. * The H3K9me2 signal derives from the presence of residual AdoMet bound to G9a and GLP during expression and purification.

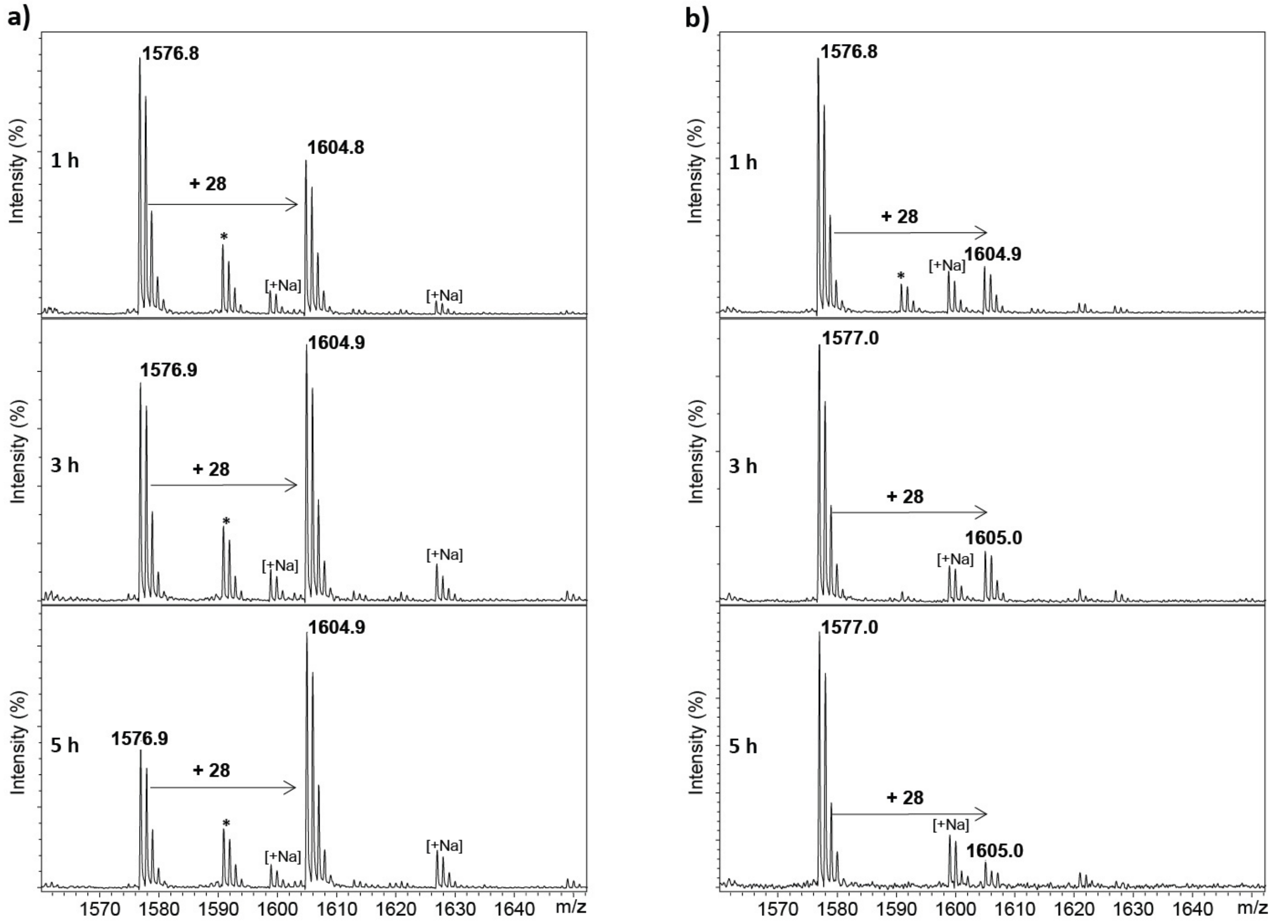


**Figure S8**. MALDI-TOF MS data showing (**a**) G9a (2 µM) catalyzed ethylation of H3K9me (40 µM) in the presence of AdoSeEth (1 mM) after incubation for 1 h (top panel), 3 h (middle panel), 5 h (bottom panel) at 37 °C; (**b**) GLP (2 µM) catalyzed traces amount of ethylation for H3K9me (40 µM) in the presence of AdoSeEth (1 mM) after incubation for 1 h (top panel), 3 h (middle panel), 5 h (bottom panel) at 37 °C. * The H3K9me2 signal derives from the presence of residual AdoMet bound to G9a and GLP during expression and purification.

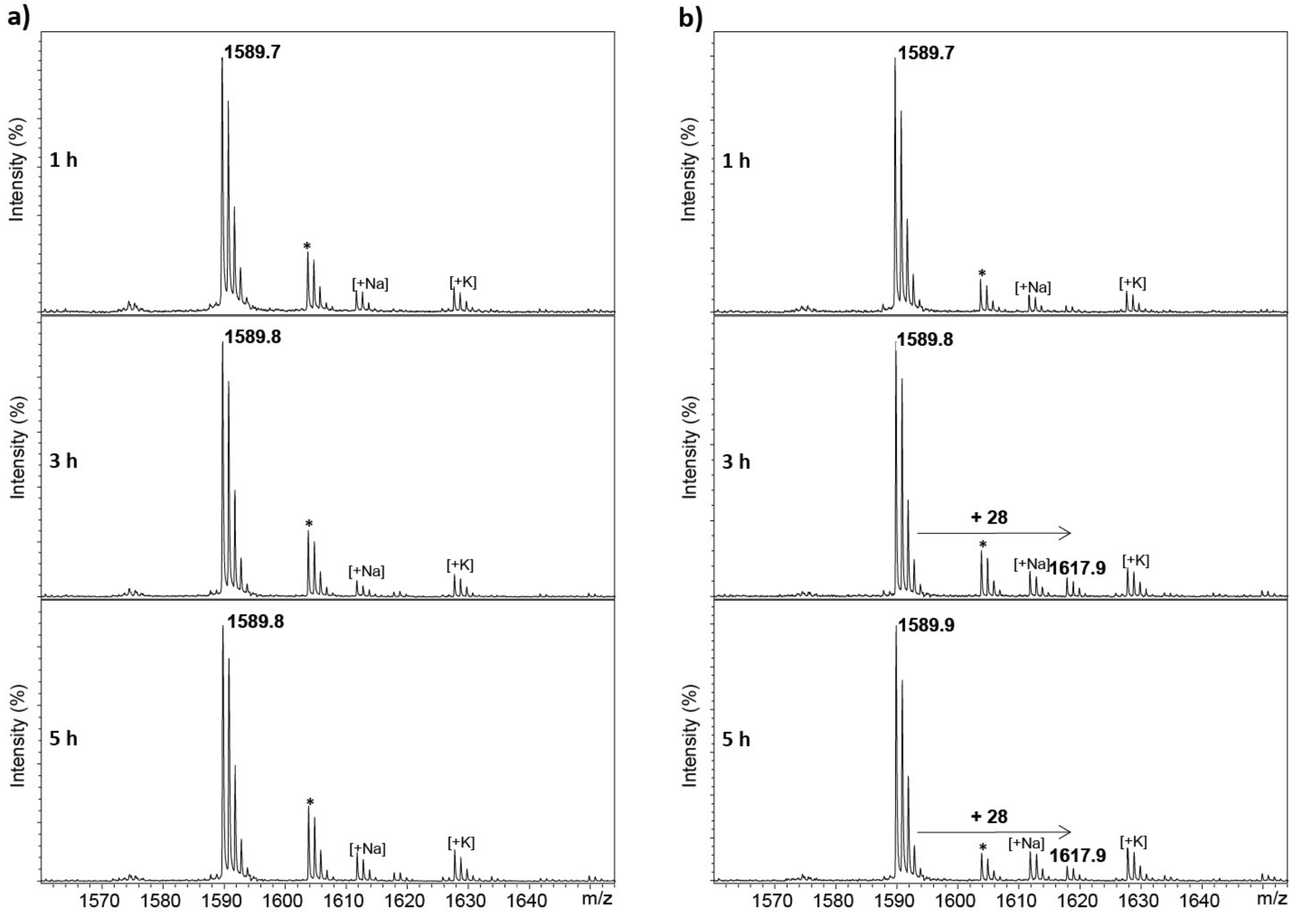


**Figure S9**. MALDI-TOF MS data showing (**a**) a lack of ethylation for H3K9me2 (40 μM) in the presence of G9a (2 μM) and AdoEth (1 mM) after incubation for 1 h (top panel), 3 h (middle panel), 5 h (bottom panel) at 37 °C; (**b**) ) a lack of ethylation for H3K9me2 (40 μM) in the presence of GLP (2 μM) and AdoEth (1 mM) after incubation for 1 h (top panel), and only trace amounts of ethylation product after 3 h (middle panel), and 5 h (bottom panel) at 37 °C. * The H3K9me3 signal derives from the presence of residual AdoMet bound to G9a and GLP during expression and purification.

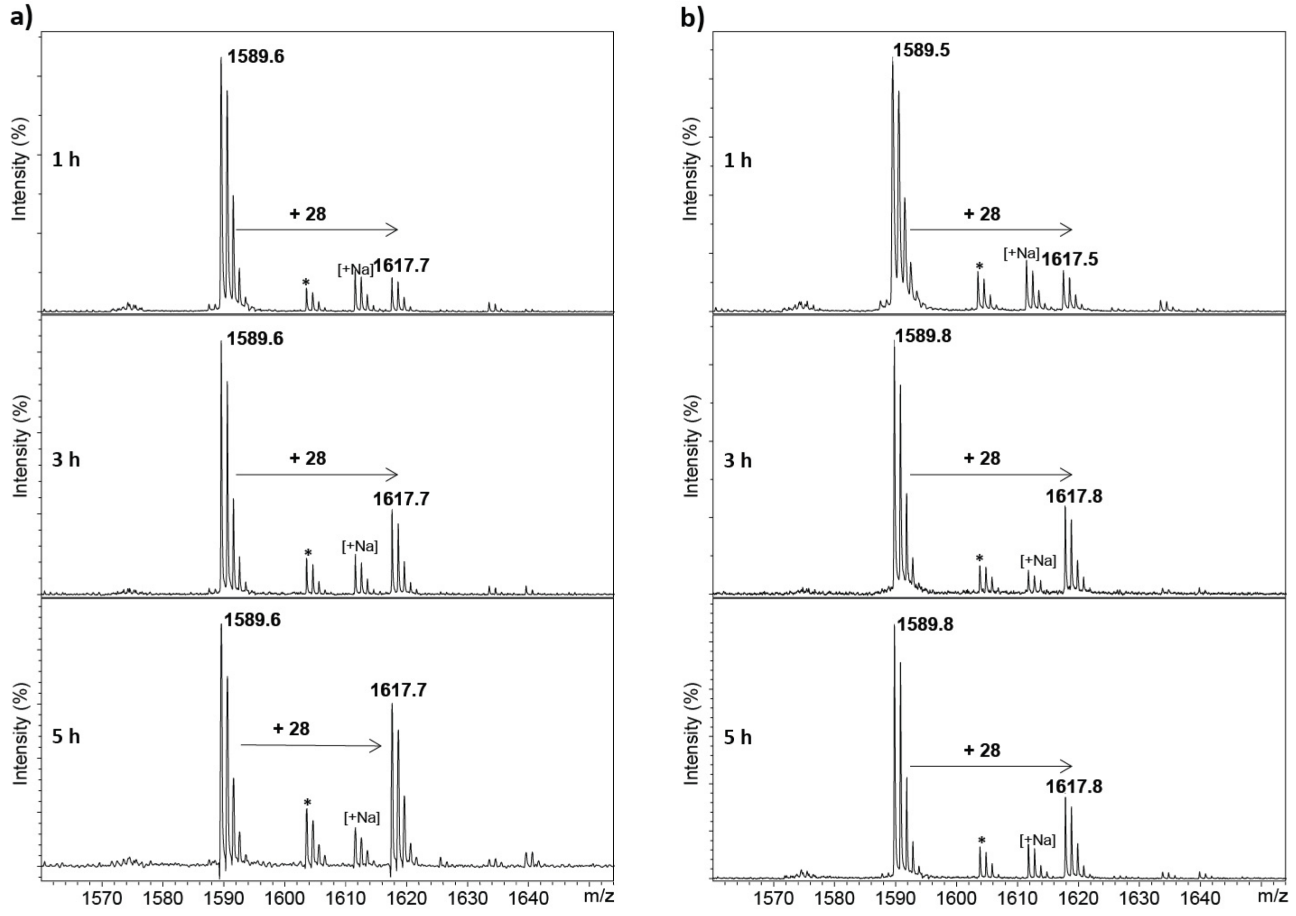


**Figure S10**. MALDI-TOF MS data showing (**a**) G9a (2 µM) catalyzed ethylation of H3K9me2 (40 µM) in the presence of AdoSeEth (1 mM) after incubation for 1 h (top panel), 3 h (middle panel), 5 h (bottom panel) at 37 °C; (**b**) GLP (2 µM) catalyzed ethylation of H3K9me2 (40 µM) in the presence of AdoSeEth (1 mM) after incubation for 1 h (top panel), 3 h (middle panel), 5 h (bottom panel) at 37 °C. * The H3K9me3 signal derives from the presence of residual AdoMet bound to G9a and GLP during expression and purification.

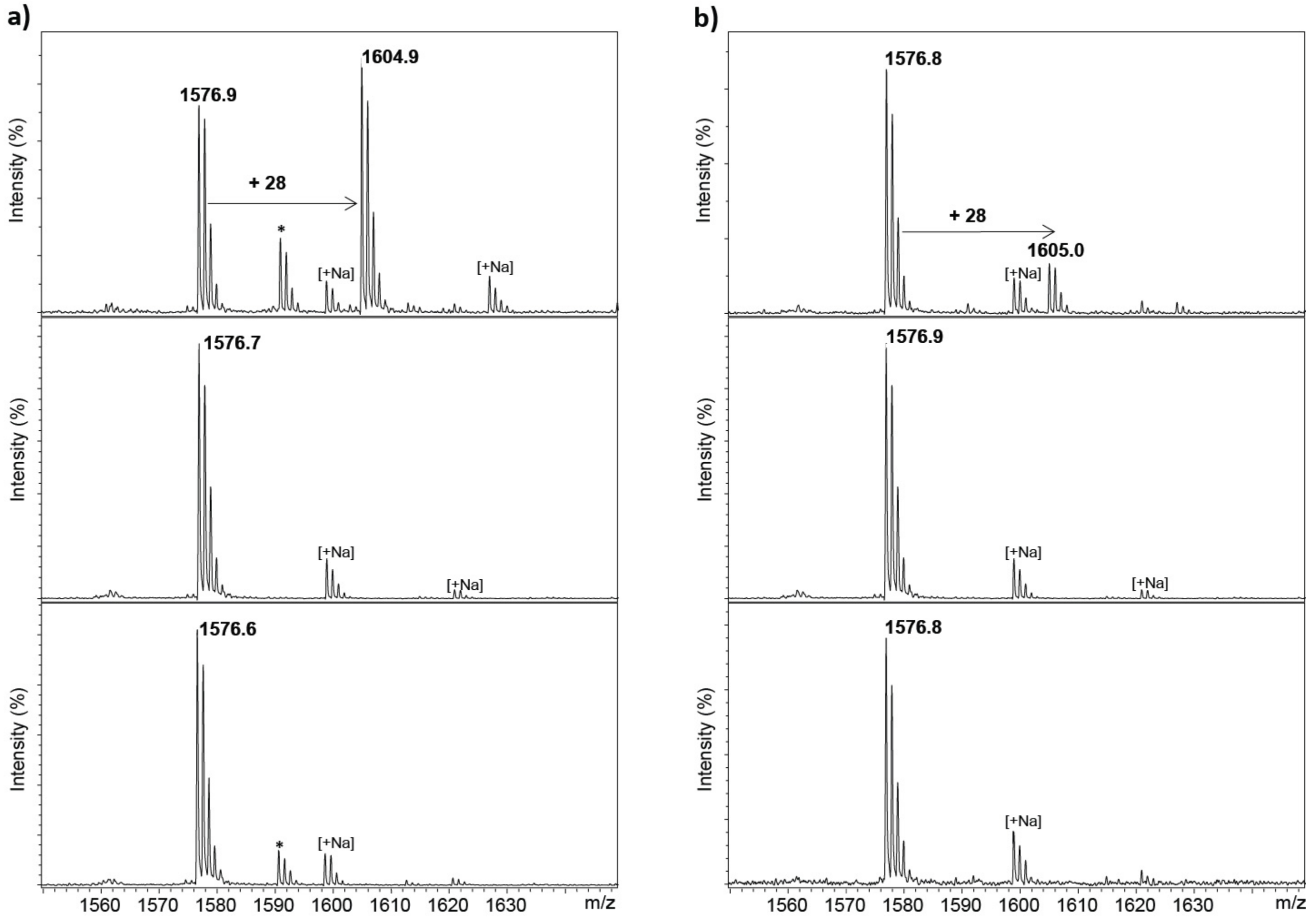


**Figure S11.** MALDI-TOF MS data showing (**a**) G9a (2 μM) catalyzed ethylation of H3K9me (40 μM) in the presence of AdoSeEth (1 mM) after 3 h (top panel) at 37 °C. Control reaction in the absence of G9a (middle panel). Control reaction in the absence of AdoSeEth (bottom panel). (**b**) GLP (2 μM) catalyzed ethylation of H3K9me (40 μM) in the presence of AdoSeEth (1 mM) after 3 h (top panel) at 37 °C. Control reaction in the absence of GLP (middle panel). Control reaction in the absence of AdoSeEth (bottom panel). * The H3K9me2 signal derives from the presence of residual AdoMet bound to G9a and GLP during expression and purification.

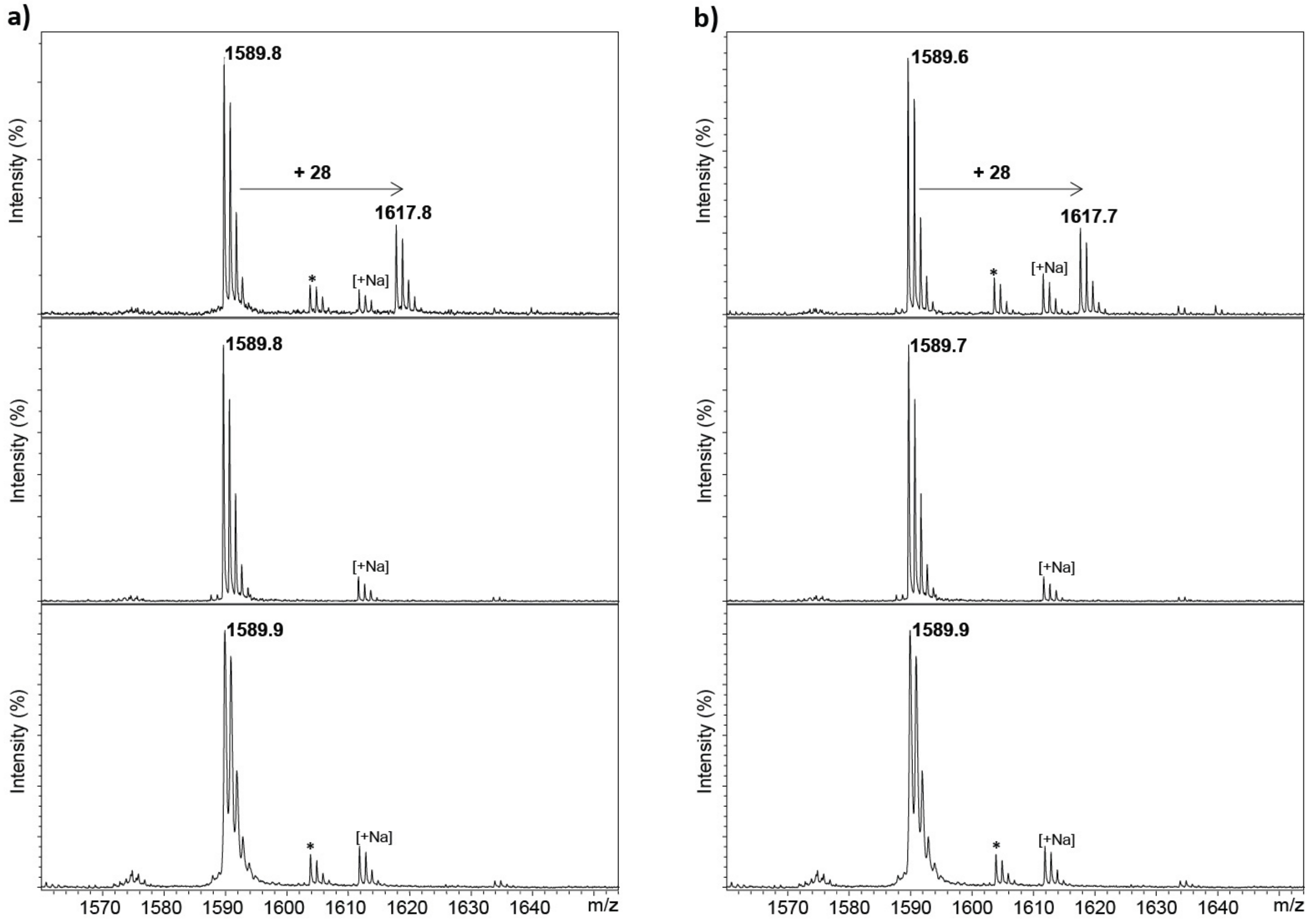


**Figure S12.** MALDI-TOF MS data showing (**a**) G9a (2 µM) catalyzed ethylation of H3K9me2 (40 µM) in the presence of AdoSeEth (1 mM) after 3 h (top panel) at 37 °C. Control reaction in the absence of G9a (middle panel). Control reaction in the absence of AdoSeEth (bottom panel). (**b**) GLP (2 µM) catalyzed ethylation for H3K9me2 (40 µM) in the presence of AdoSeEth (1 mM) after 3 h (top panel) at 37 °C. Control reaction in the absence of GLP (middle panel). Control reaction in the absence of AdoSeEth (bottom panel). * The H3K9me3 signal derives from the presence of residual AdoMet bound to G9a and GLP during expression and purification.

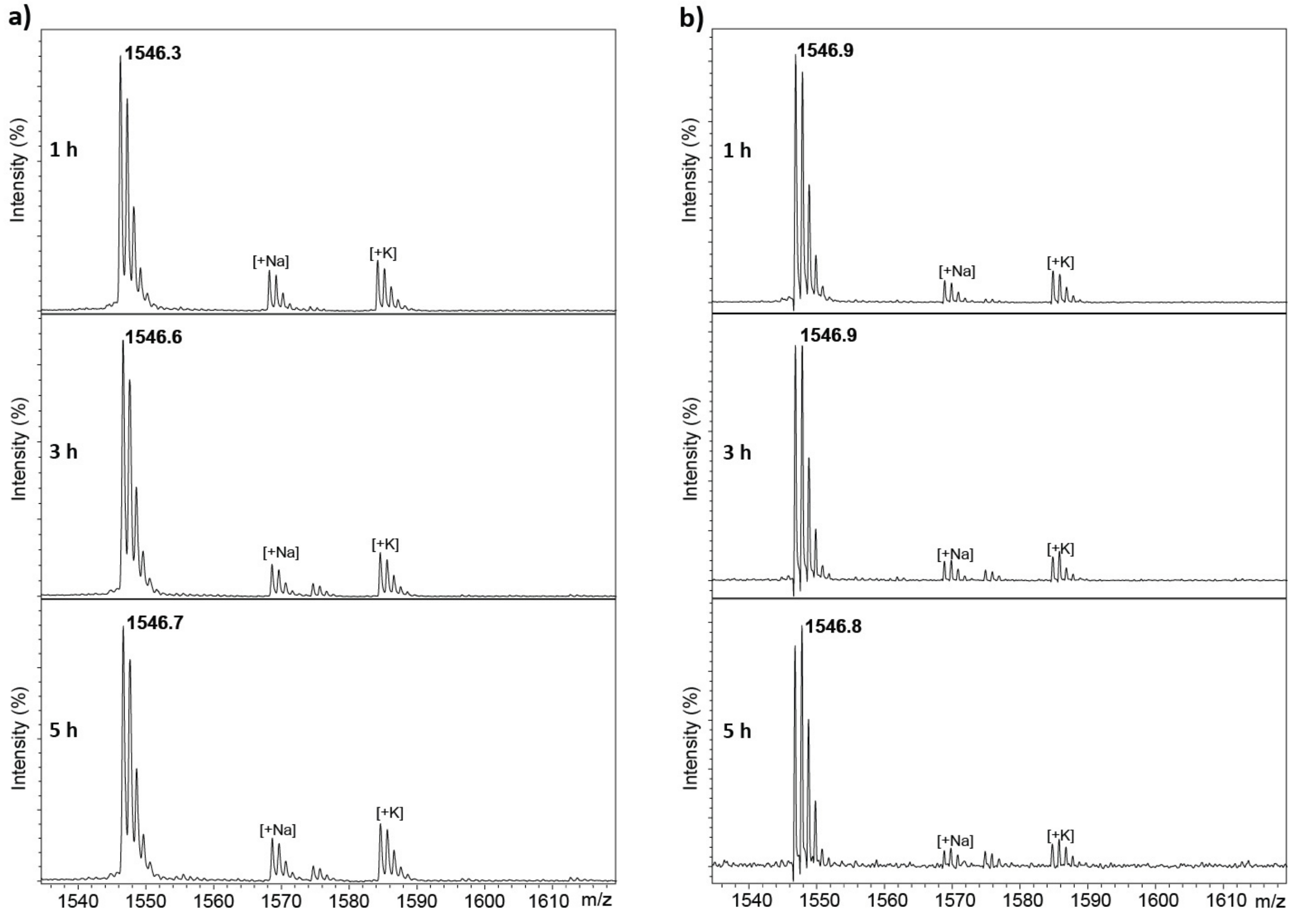


**Figure S13**. MALDI-TOF MS analysis showing a lack of ethylation for (**a**) H3Orn9 (40 µM) in the presence of G9a (2 µM) and AdoEth (1 mM) after incubation for 1 h (top panel), 3 h (middle panel), and 5 h (bottom panel) at 37 °C; (**b**) H3Orn9 (40 µM) in the presence of GLP (2 µM) and AdoEth (1 mM) after incubation for 1 h (top panel), 3 h (middle panel), and 5 h (bottom panel) at 37 °C.

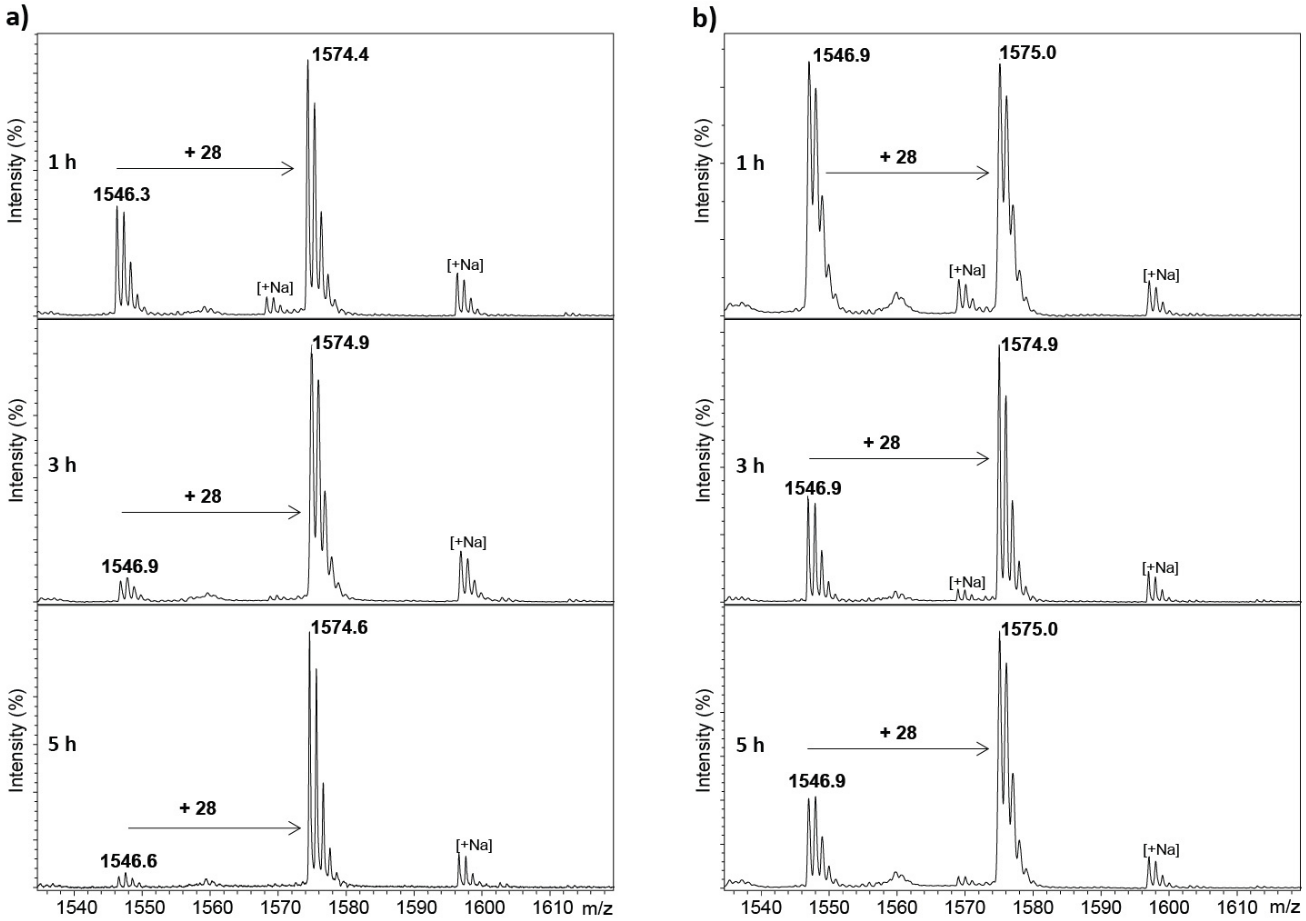


**Figure S14**. MALDI-TOF MS data showing (**a**) G9a (2 µM) catalyzed ethylation of H3Orn9 (40 µM) in the presence of AdoSeEth (1 mM) after incubation for 1 h (top panel), 3 h (middle panel), 5 h (bottom panel) at 37 °C; (**b**) GLP (2 µM) catalyzed ethylation of H3Orn9 (40 µM) in the presence of AdoSeEth (1 mM) after incubation for 1 h (top panel), 3 h (middle panel), 5 h (bottom panel) at 37 °C.

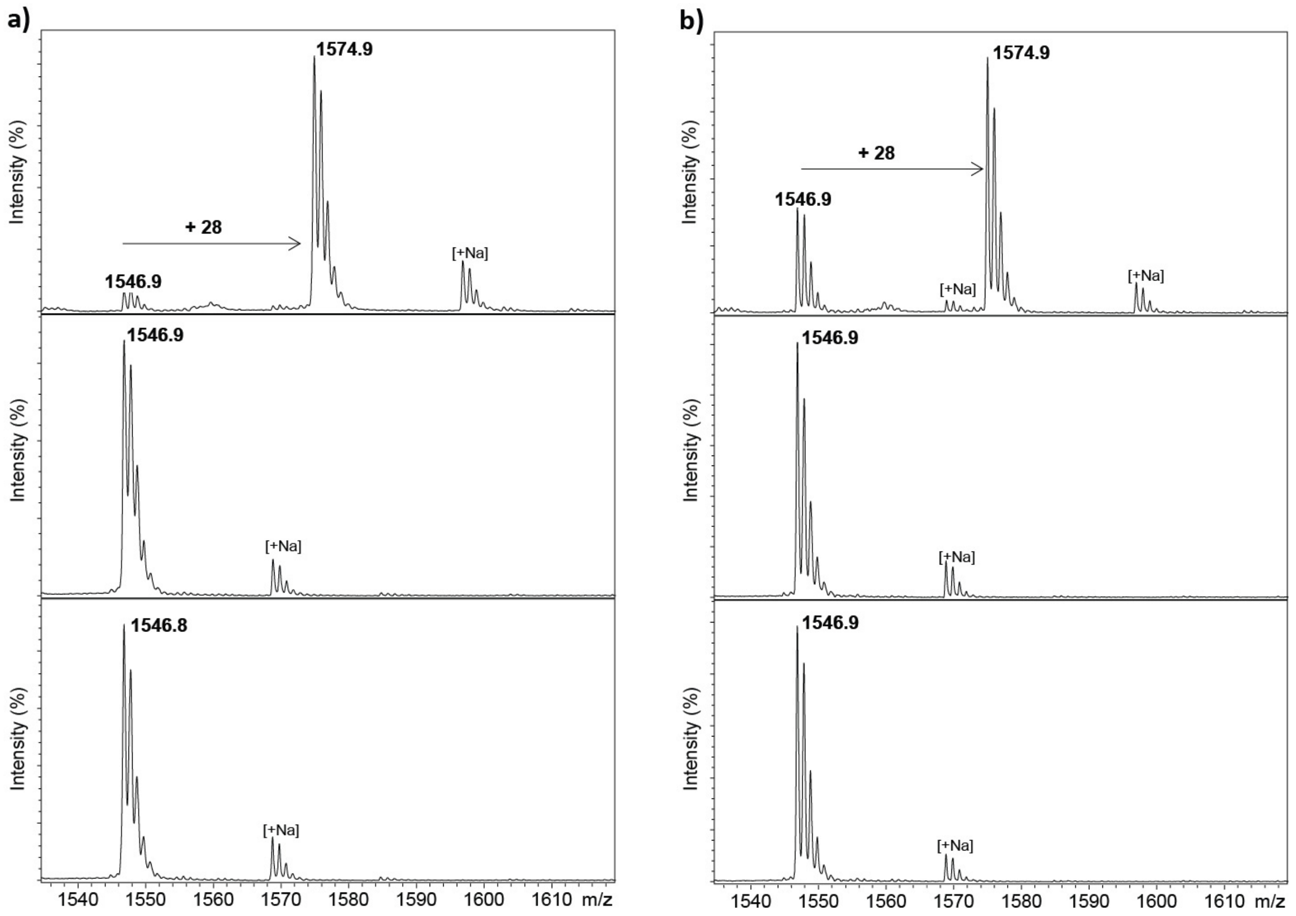


**Figure S15.** MALDI-TOF MS data showing (**a**) G9a (2 µM) catalyzed ethylation of H3Orn9 (40 µM) in the presence of AdoSeEth (1 mM) after 3 h (top panel) at 37 °C. Control reaction in the absence of G9a (middle panel). Control reaction in the absence of AdoSeEth (bottom panel). (**b**) GLP (2 µM) catalyzed ethylation of H3Orn9 (40 µM) in the presence of AdoSeEth (1 mM) after 3 h (top panel) at 37 °C. Control reaction in the absence of GLP (middle panel). Control reaction in the absence of AdoSeEth (bottom panel).

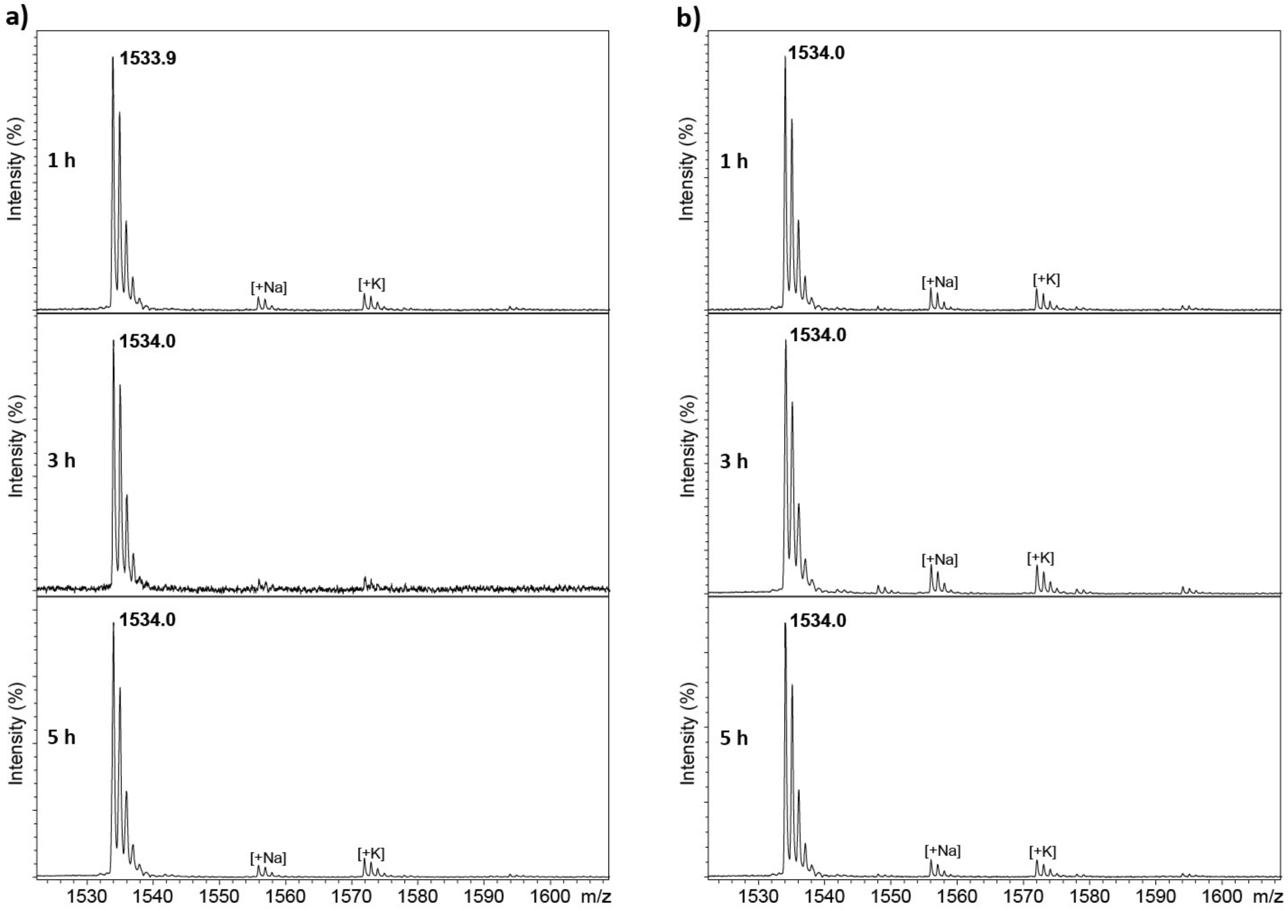


**Figure S16**. MALDI-TOF MS analysis showing a lack of ethylation for (**a**) H3Dab9 (40 µM) in the presence of G9a (2 µM) and AdoEth (1 mM) after incubation for 1 h (top panel), 3 h (middle panel), and 5 h (bottom panel) at 37 °C; (**b**) H3Dab9 (40 µM) in the presence of GLP (2 µM) and AdoEth (1 mM) after incubation for 1 h (top panel), 3 h (middle panel), and 5 h (bottom panel) at 37 °C.

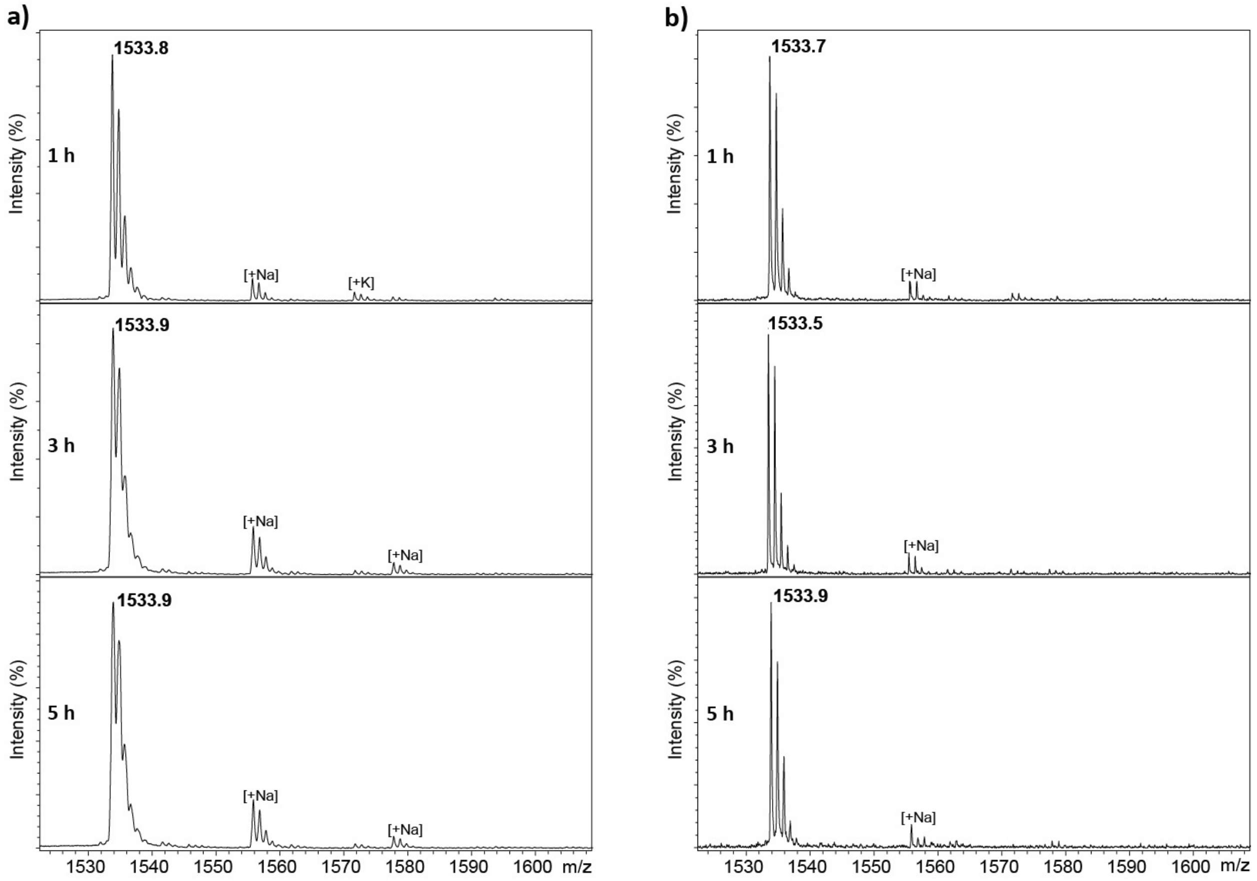


**Figure S17**. MALDI-TOF MS analysis showing a lack of ethylation for (**a**) H3Dab9 (40 µM) in the presence of G9a (2 µM) and AdoSeEth (1 mM) after incubation for 1 h (top panel), 3 h (middle panel), and 5 h (bottom panel) at 37 °C; (**b**) H3Dab9 (40 µM) in the presence of GLP (2 µM) and AdoSeEth (1 mM) after incubation for 1 h (top panel), 3 h (middle panel), and 5 h (bottom panel) at 37 °C.

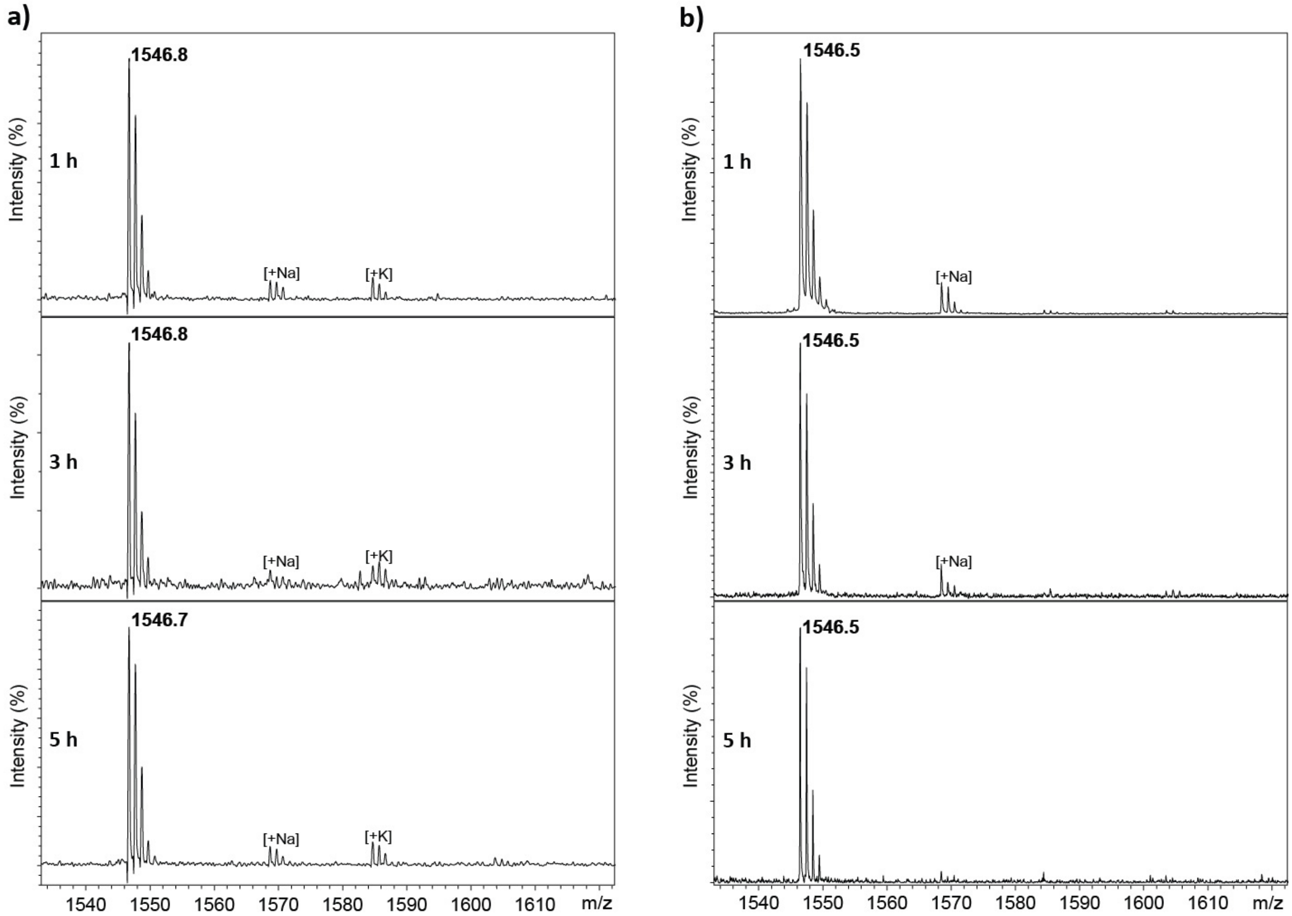


**Figure S18**. MALDI-TOF MS analysis showing a lack of ethylation for (**a**) H3Orn4 (40 µM) in the presence of SETD7 (2 µM) and AdoEth (1 mM) after incubation for 1 h (top panel), 3 h (middle panel), and 5 h (bottom panel) at 37 °C; (**b**) H3Orn4 (40 µM) in the presence of SETD7 (2 µM) and AdoSeEth (1 mM) after incubation for 1 h (top panel), 3 h (middle panel), and 5 h (bottom panel) at 37 °C.

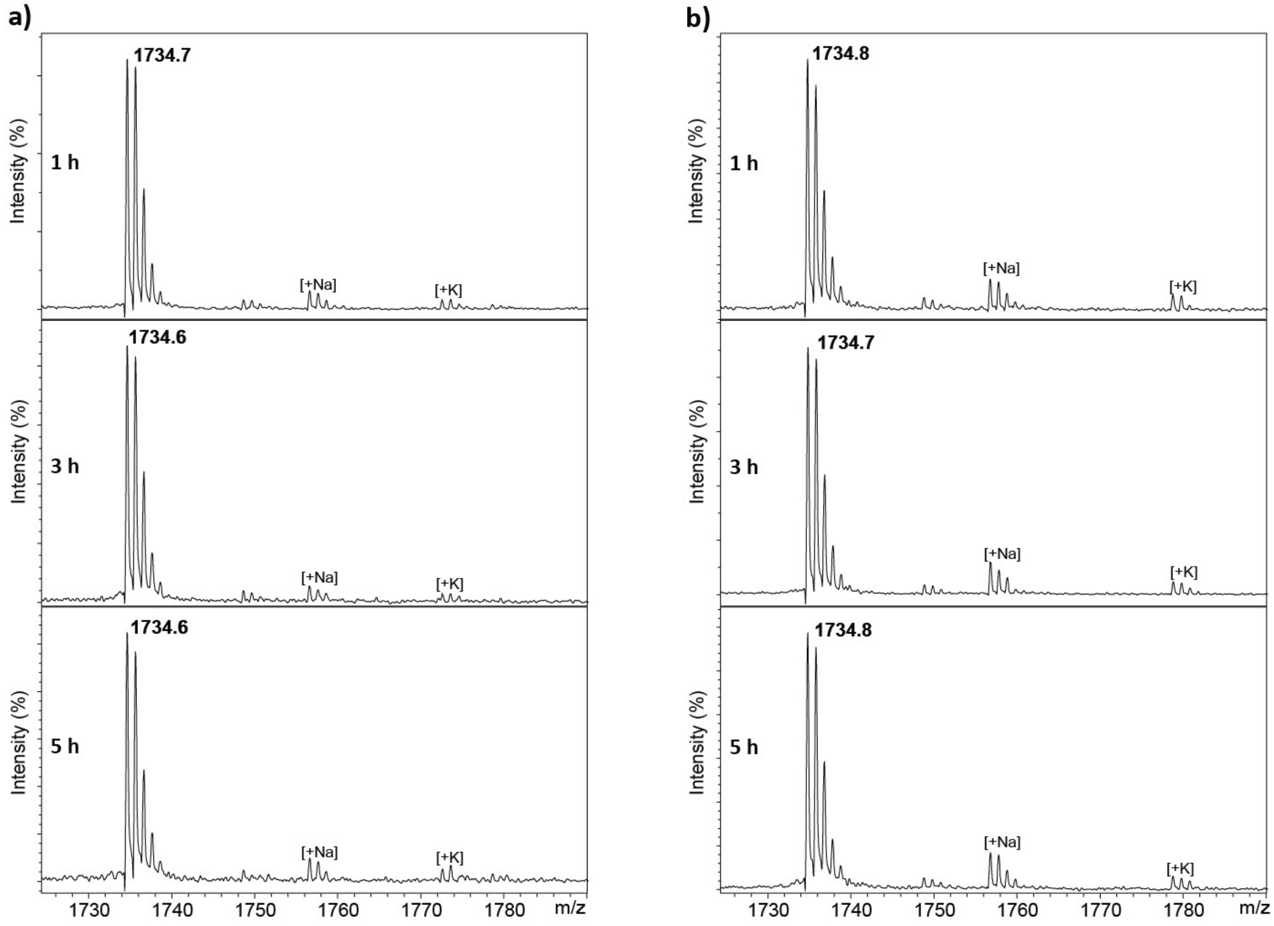


**Figure S19**. MALDI-TOF MS analysis showing a lack of ethylation for (**a**) H4Orn20 (40 µM) in the presence of SETD8 (2 µM) and AdoEth (1 mM) after incubation for 1 h (top panel), 3 h (middle panel), and 5 h (bottom panel) at 37 °C; (**b**) H4Orn20 (40 µM) in the presence of SETD8 (2 µM) and AdoSeEth (500 µM) after incubation for 1 h (top panel), 3 h (middle panel), and 5 h (bottom panel) at 37 °C.

## 2. Enzyme kinetics analyses

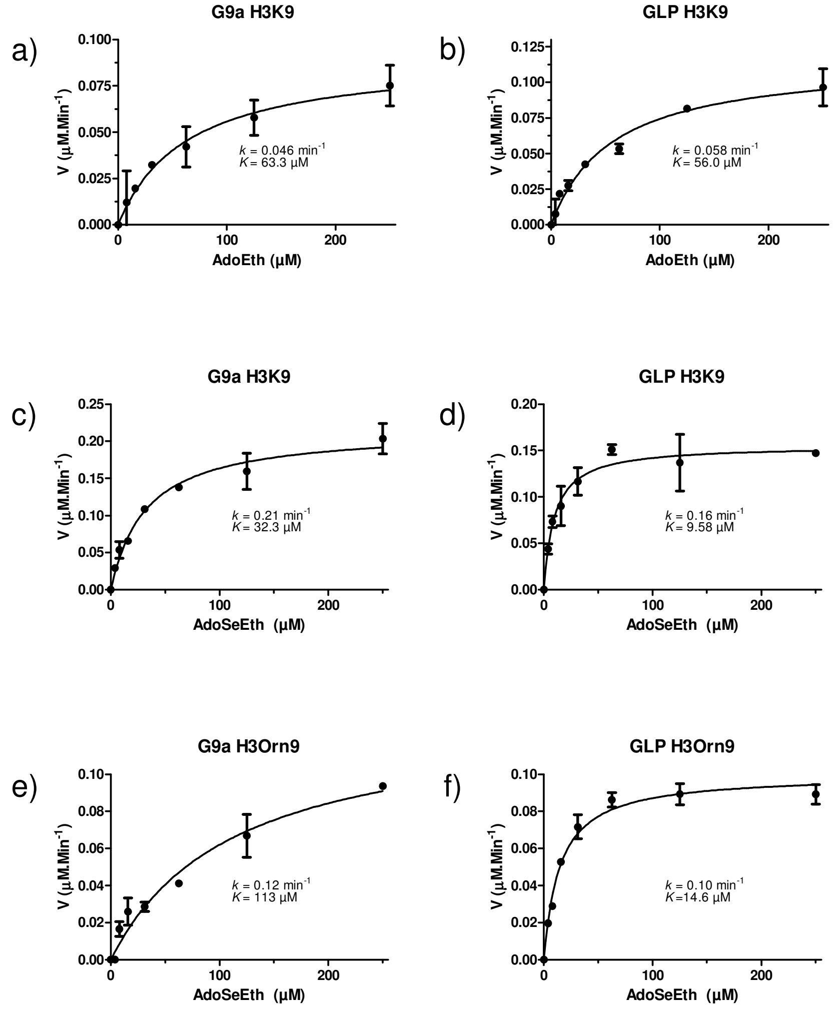


**Figure S20**. Kinetics analyses for a) G9a (2 µM) catalyzed ethylation of H3K9 (25 µM) in the presence of AdoEth (0-250 µM); b) GLP (2 µM) catalyzed ethylation of H3K9 (25 µM) in the presence of AdoEth (0-250 µM); c) G9a (1 µM) catalyzed ethylation of of H3K9 (25 µM) in the presence of AdoSeEth (0-250 µM); d) GLP (1 µM) catalyzed ethylation of H3K9 (25 µM) in the presence of AdoSeEth (0-250 µM); e) G9a (1 µM) catalyzed ethylation of H3Orn9 (25 µM) in the presence of AdoSeEth (0-250 µM); f) GLP (1 µM) catalyzed ethylation of H3Orn9 (25 µM) in the presence AdoSeEth (0-250 µM).

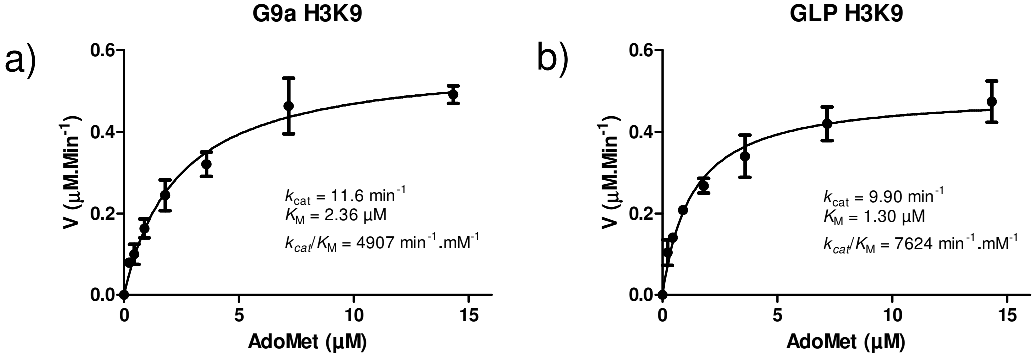


**Figure S21.** Michaelis-Menten curves for a) G9a (50 nM) catalyzed methylation of H3K9 (10 μM) in the presence of AdoMet (0-15 μM); b) GLP (50 nM) catalyzed methylation of H3K9 (10 μM) in the presence of AdoMet (0-15 μM).

## 3. Computational figures

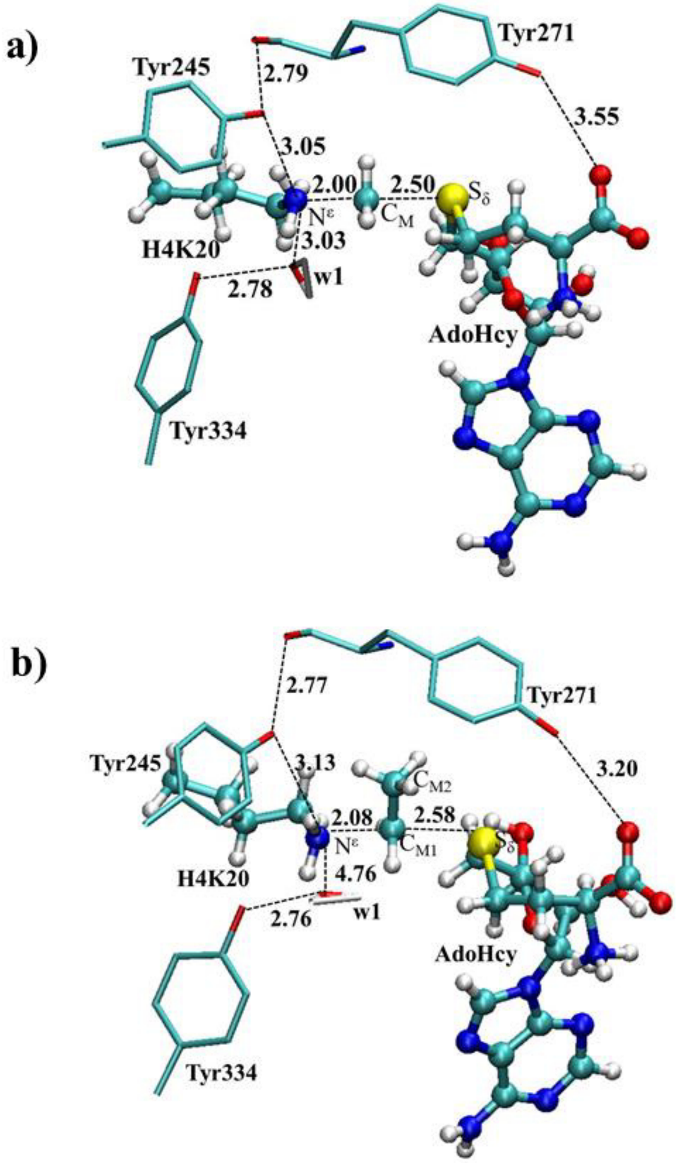


**Figure S22.** a) Representative active-site structure near the transition state for the first methylation in SETD8 obtained from the QM/MM MD simulations. b) Representative active-site structure near the transition state for the first ethylation in SETD8.

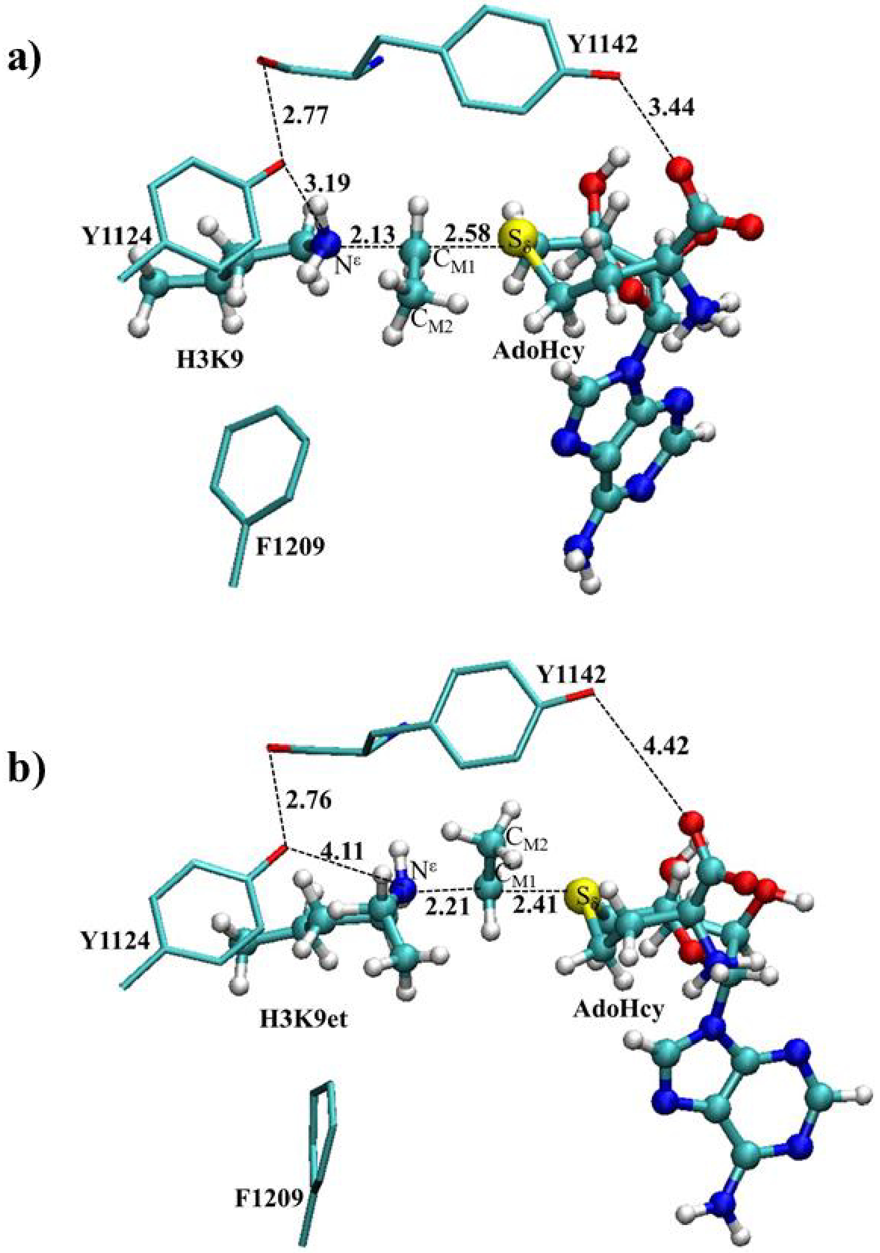


**Figure S23.** a) Representative active-site structure near the transition state for the first ethylation in GLP obtained from the QM/MM MD simulations. b) Representative active-site structure near the transition state for the second ethylation in GLP.

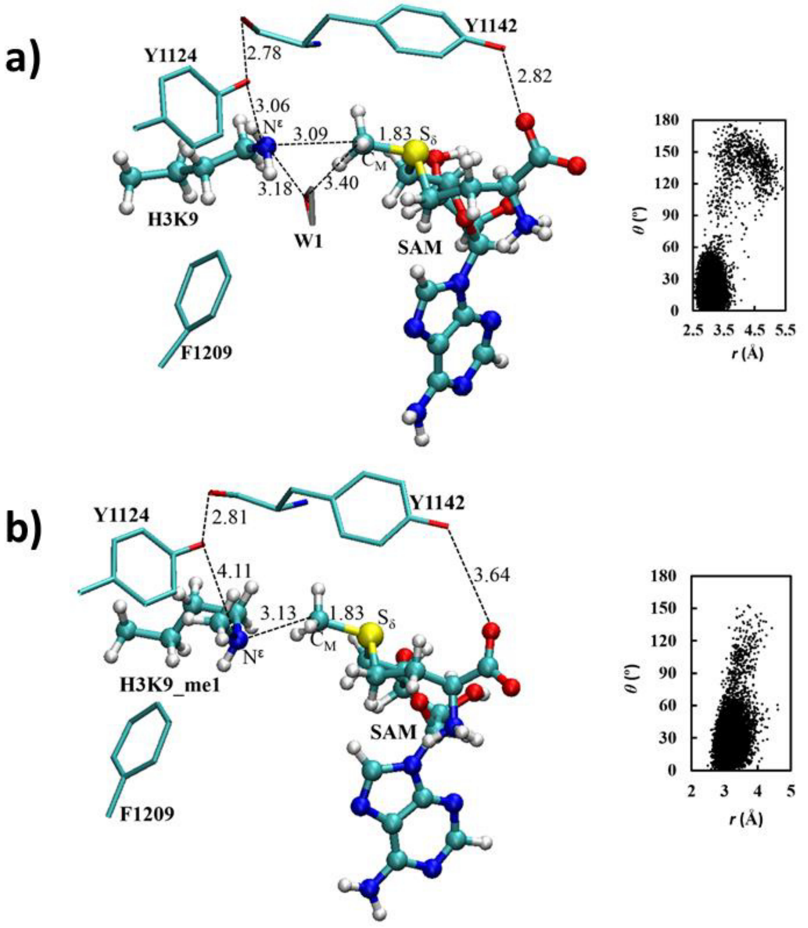


**Figure S24.** a) Representative active-site structure of the reactant complex of GLP for the first methylation along with the r($C_M \cdots N^{\varepsilon}$) and θ distribution map obtained from the QM/MM MD simulations. b) Representative active-site structure of the reactant complex of GLP for the second methylation.
